# Silencing mitochondrial gene expression in living cells

**DOI:** 10.1126/science.adr3498

**Published:** 2025-07-31

**Authors:** Luis Daniel Cruz-Zaragoza, Drishan Dahal, Mats Koschel, Angela Boshnakovska, Aiturgan Zheenbekova, Mehmet Yilmaz, Marcel Morgenstern, Jan-Niklas Dohrke, Julian Bender, Anusha Valpadashi, Kristine A Henningfeld, Silke Oeljeklaus, Laura Sophie Kremer, Mirjam Breuer, Oliver Urbach, Sven Dennerlein, Michael Lidschreiber, Stefan Jakobs, Bettina Warscheid, Peter Rehling

**Affiliations:** 1 Department of Cellular Biochemistry, University Medical Center Göttingen, D-37073 Göttingen, Germany; 2 Department of Molecular Biology, Max Planck Institute for Multidisciplinary Sciences, Göttingen, Germany; 3 Faculty of Chemistry and Pharmacy, Biochemistry II, Theodor Boveri-Institute, Biocenter, University of Würzburg, D-97074 Wuerzburg, Germany; 4 Department of NanoBiophotonics, Max Planck Institute for Multidisciplinary Sciences, Göttingen, Germany; 5 Clinic of Neurology, University Medical Center Göttingen, Göttingen, Germany; 6 Fraunhofer Institute for Translational Medicine and Pharmacology, Translational Neuroinflammation and Automated Microscopy, Göttingen, Germany; 7 Cluster of Excellence “Multiscale Bioimaging: from Molecular Machines to Networks of Excitable Cells” (MBExC), University of Göttingen, Germany; 8 Max Planck Institute for Multidisciplinary Sciences, D-37077, Göttingen, Germany

## Abstract

Mitochondria fulfill central functions in metabolism and energy supply. They express their own genome, which encodes key subunits of the oxidative phosphorylation system. However, central mechanisms underlying mitochondrial gene expression remain enigmatic. A lack of suitable technologies to target mitochondrial protein synthesis in cells has limited experimental access. Here, we silenced the translation of specific mitochondrial mRNAs in living human cells by delivering synthetic peptide-morpholino chimeras. This approach allowed us to perform a comprehensive temporal monitoring of cellular responses. Our study provides insights into mitochondrial translation, its integration into cellular physiology, and provides a strategy to address mitochondrial gene expression in living cells. The approach can potentially be used to analyze mechanisms and pathophysiology of mitochondrial gene expression in a range of cellular model systems.

Human mitochondrial DNA (mtDNA) encodes two rRNAs, 22 tRNAs, and eleven mRNAs, two of which are bicistronic (1). Translation of mitochondrial mRNAs occurs on membrane-associated ribosomes that facilitate insertion of the translation products into the inner mitochondrial membrane in cooperation with the OXA1L insertase (2, 3). Defects in the synthesis of mitochondria-encoded proteins are linked to many severe human disorders (4–7). Central aspects of mitochondrially encoded gene expression mechanisms remain unresolved. For instance, we lack mechanistic insights into how mitochondrial and nuclear gene expression are coordinated to enable the formation of the oxidative phosphorylation (OXPHOS) system from subunits of dual genetic origin (8, 9). Despite initial attempts to reconstitute mitochondrial translation in vitro (10) and to assess its dynamics by ribosome profiling (11), we have a limited understanding of the proteins that coordinate gene expression processes in mitochondria and direct mRNAs for translation (12). Furthermore, we do not know how cells cope with challenges to the synthesis of specific mitochondria-encoded polypeptides over time. Unlike nuclear DNA, mtDNA cannot be gene-edited via CRISPR because transporting RNAs into mitochondria is not an established approach (13, 14). Instead, targeted gene editing of mtDNA has only recently become possible using bacterial cytidine deaminases (15–18). However, approaches that allow selective protein expression silencing in mitochondria in a simple and kinetically resolved manner in cells are still not available.

Here, we developed a simple and efficient approach to selectively target and silence individual mitochondrial mRNAs in cells. Our method used chimeras composed of a mitochondrial targeting peptide and a polymorpholino oligonucleotide, which were synthesized using click chemistry and imported into mitochondria to block translation. This approach allowed us to target multiple mRNAs simultaneously and apply it to a wide range of cell types.

## Design of highly efficient chimera to silence mitochondrial translation

Previously, we established a system to silence mitochondrial gene expression (19), in which morpholinos directed against mitochondrial mRNAs are imported into purified mitochondria. For this, morpholinos are linked to a mitochondrial precursor protein that acts as a targeting module directing the morpholino to the matrix. However, the approach is limited in scope because it requires the ability to carry out import and subsequent gene expression analyses in isolated mitochondria in vitro. In addition, challenges to mitochondrial gene expression cannot be analyzed in the context of a cellular environment and cannot be applied to understand mitochondrial-nuclear crosstalk. Despite extensive efforts, the previously designed precursor protein-morpholino chimeras were ineffective in modulating mitochondrial translation in cells. Therefore, we designed an alternative chimera in which the precursor targeting module of the chimera was substituted by a presequence peptide to target mitochondrial mRNAs in living cells.

**Fig. 1:**
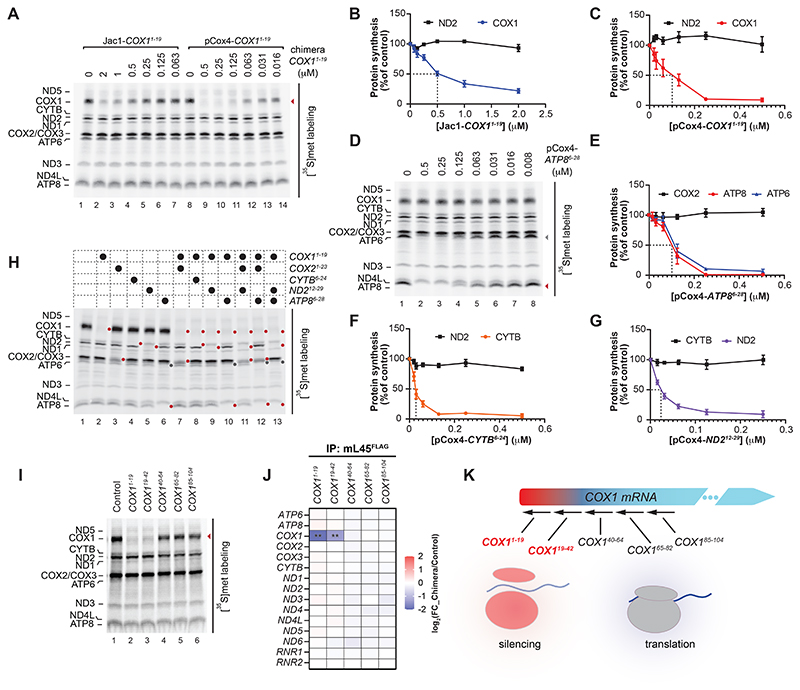
Gene silencing by peptide-morpholino chimeras is more efficient and versatile. See also fig. S1. (**A**) Jac1-*COX1*^*1-19*^ and pCox4-*COX1*^*1-19*^ were imported into isolated mitochondria from HEK293T cells. After import, mitochondria were re-isolated, subjected to in vitro translation in the presence of [^35^S]methionine, and newly synthesized polypeptides analyzed by SDS-PAGE followed by digital autoradiography. (**B** and **C**) Quantification of in organello COX1 silencing effect by Jac1-*COX1*^*1-19*^ chimera titration (IC_50_ ~ 0,5 μM; n=4, mean ± SEM) and pCox4-*COX1*^*1-19*^ chimera titration (IC_50_ ~ 0,1 μM; n=4, mean ± SEM). (**D** and **E**) Titration of pCox4-*ATP8*^*6-28*^ chimera effect on protein translation in HEK293T cell mitochondria (as in A) and quantification (IC_50_ ~ 0,1 μM; n=4, mean ± SEM). ATP6 synthesis was also downregulated, confirming coupled synthesis. (**F** and **G**) Quantification of in organello silencing by CYTB (pCox4-*CYTB*^*6-24*^ chimera, IC_50_ ~ 0,015 μM; n=4, mean ± SEM) and ND2 (pCox4-*ND2*^*12-29*^ chimera, IC_50_ ~ 0,03 μM; n=4, mean ± SEM) in HEK293T mitochondria. (**H**) Silencing the gene expression of one, two, and three mitochondrial-encoded mRNAs in isolated HEK293T mitochondria. Targeted protein indicated by red dot; ATP6 upon ATP8 silencing indicated by gray dot. (**I**) In organello silencing effect of chimeras targeting different *COX1* mRNA 5’ region. Chimera binding region in the transcript are presented as superscript. (**J**) Heatmap of mtDNA-encoded mRNAs and rRNAs (*RNR1* and *RNR2*) amounts associated with the ribosome upon treatment with COX1-targeting chimeras from (I), compared to the Control (pCox4) (mean, n=3). Statistical significance was determined by multiple t test using the Holm-Sidak method, with alpha=0.05 (^**^, padj<0.01). Non-significant differences are not indicated. Mitochondria isolated from mL45^FLAG^-expressing HEK293T cells treated with chimeras used in (I), FLAG-IP was performed, RNA purified from eluate fractions, and analyzed by nanoString. *COX1* mRNA binding to the ribosome was significantly reduced after treatment with *COX1*^*1-19*^ and *COX1*^*19-42*^. FC, fold change. (**K**) Schematic representation of chimera’s mechanism of action in mitochondria.

After testing various presequence peptides, we selected the Cox4 presequence (pCox4^1-25^) from *Saccharomyces cerevisiae*. Chimeras were synthesized by a click chemistry reaction linking the pCox4-N_3_ with the phosphorodiamidate morpholino oligomer (MO) modified with a cyclooctyne group (Fig. S1A). We initially compared the translation-blocking efficiency of pCox4-*COX1*^*1-19*^ to the previously used precursor protein Jac1-*COX1*^*1-19*^ chimera (19). Chimeras were imported into purified mitochondria, and translation was monitored by [^35^S]methionine labeling of mitochondrial translation products. While both chimeras led to a robust and specific reduction of COX1 translation in purified mitochondria, the pCox4-*COX1*^*1-19*^ chimera (IC_50_~0.1 µM) was five times more efficient than the Jac1-*COX1*^*1-19*^ chimera (IC_50_~0.5 µM) (Fig. 1, A-C). Peptide-chimeras targeting the other human mitochondrial mRNAs (except ND6) displayed similar translation-blocking activity in purified mitochondria (fig. S1, B-F). We efficiently blocked the translation of the bicistronic *ATP8/ATP6* (Fig. 1, D and E), *CYTB*, and *ND2* (Fig. 1, F and G) mRNAs. The peptide-based chimera could also be applied to purified mouse mitochondria to block COX1 synthesis (fig. S1G). Because peptide-morpholino chimeras efficiently blocked translation at nanomolar concentrations, we assessed whether they could be used to target multiple mRNAs in parallel. We imported various chimera combinations targeting different mRNAs into mitochondria and evaluated translation by [^35^S]methionine labeling. Indeed, we could specifically silence three different mRNAs simultaneously (Fig. 1H). Morpholinos have been used to inhibit cytosolic translation by blocking the cognate mRNA interaction with the ribosome (20). To elucidate their mechanism of action in mitochondria, we evaluated the silencing capacity of different chimeras targeting various positions in the *COX1* mRNA 5’ region. Effective silencing in isolated mitochondria was only observed if the chimera targeted the region 1-42 (Fig. 1I). When we isolated mitochondrial ribosomes via mL45^FLAG^ under these conditions and assessed the transcripts in the eluates by nanoString, the *COX1* mRNA association to the ribosome was specifically reduced upon treatment with *COX1*^*1-19*^ and *COX1*^*19-42*^ chimeras but not with chimera targeting the mRNA downstream of this region (Fig. 1J). Thus, chimeras consisting of the Cox4 presequence and a polymorpholino (pCox4-MO) targeting mitochondrial mRNAs efficiently block translation by inhibiting ribosome-mRNA interaction and, consequently, translation (Fig. 1K and fig. S1, H-I).

## Silencing mitochondrial gene expression in living cells

**Fig. 2:**
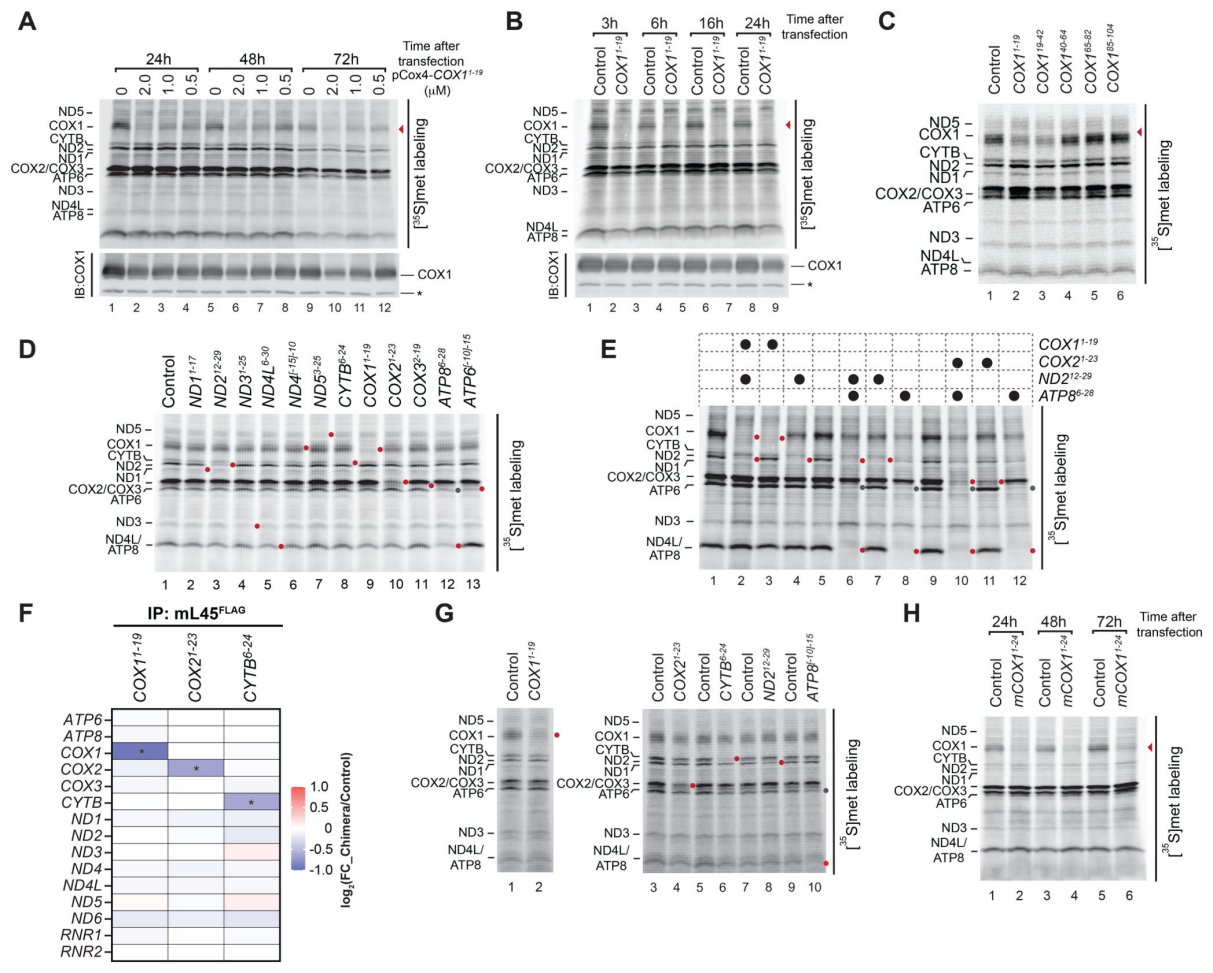
Silencing of mtDNA-encoded genes in living cells. See also fig. S2. (**A**) HEK293T cells were transfected with freshly synthesized pCox4-*COX1*^*1-19*^ at indicated concentrations. No chimera was added to the control (0). 24, 48, and 72 hours post transfection, mitochondrial translation products were metabolically labeled with [^35^S]methionine. COX1 signal indicated by arrowhead. Steady-state levels of COX1 in cell lysate were determined by immunoblot (IB). (**B**) HEK293T cells were transfected with pCox4-*COX1*^*1-19*^ for 3, 6, 16, and 24 hours and silencing confirmed as in (A). COX1 signal is indicated by an arrowhead. (**C**) Silencing effect of different chimeras targeting *COX1* mRNA 5’ region in living cells. Chimera binding region in the transcript presented as superscript. (**D**) Chimera targeting all mtDNA-encoded genes (except ND6) were transfected in HEK293T cells for 24 hours, followed by [^35^S]methionine labeling of mitochondrial translation products. Targeted protein indicated by red dot; ATP6 upon ATP8 silencing indicated by gray dot. (**E**) Simultaneous silencing of two mtDNA-encoded mRNAs in HEK293T cells. (**F**) Heatmap representing the amount of mtDNA-encoded mRNAs (mt-mRNA) and rRNAs (mt-rRNA, *RNR1* and *RNR2*) associated to the mitochondrial ribosome upon treatment with *COX1*-, *COX2*-, and *CYTB*-targeting chimeras compared to control (pCox4). mL45^FLAG^-expressing HEK293T cells were treated with chimeras silencing COX1, COX2, and CYTB for 4, 16, and 16 hours, respectively. Ribosomes were purified by FLAG-IP and associated RNAs detected in the eluate by nanoString (mean, n=3). FC, fold change. Statistical significance was determined by multiple t test using the Holm-Sidak method, with alpha=0.05 (^*^, padj<0.05). Non-significant differences are not indicated. (**G**) Downregulation of mitochondrial gene expression in human iPSC-derived cardiomyocytes treated for 24 hours with indicated chimeras, and (**H**) mouse hepatocytes (AML12 cell line) treated with pCox4-m*COX1*^*1-24*^ targeting mouse *COX1* mRNA. Downregulated newly synthesized proteins are indicated by red dot. ATP6 signal reduction, upon ATP8 downregulation, is indicated with a gray dot.

To silence mitochondrial gene expression in intact mammalian cells, the chimera must be available at high concentrations, chemically stable, and easily transported into cells. In contrast to Jac1-MO, the pCox4-MO chimera met those requirements and could be efficiently transfected (fig. S2A). We monitored mitochondrial protein synthesis by [^35^S]methionine labeling in cells in the presence of emetine to inhibit cytosolic translation. The pCox4-*COX1*^*1-19*^ chimera efficiently silenced COX1 synthesis in HEK293T cells at 24-, 48-, and 72-hours post-transfection (Fig. 2A). The transfection method did not influence mitochondrial morphology and translation (fig. S2, B-D). The steady-state protein levels of COX1, assessed by immunoblot analysis, showed a gradual decrease during the treatment, with maximal reduction 72-hours post-transfection (Fig. 2A), while mtDNA abundance was unaffected (fig. S2E). COX1 translation and steady-state levels correlated with the chimera concentration in the culture media. In addition to the long-term application of chimera, we evaluated the efficiency of the translation block at short time points. A robust inhibition of COX1 translation was already apparent three hours post-transfection of pCox4-*COX1*^*1-19*^ (Fig. 2B).

The silencing effect of targeting different *COX1* mRNA 5’ regions in living cells displayed a similar pattern to that observed in isolated mitochondria (Fig. 2C). Taking advantage of the morpholino’s fluorescein isothiocyanate (FITC) fluorophore, we determined cellular localization of the chimera by mitochondria immunocapture. Like the mitochondrial outer membrane marker TOM20, the chimera was found to be enriched in the mitochondrial fraction, as expected (fig. S2F).

We generated chimeras targeting mRNAs of mtDNA-encoding subunits of OXPHOS complexes I (*ND1-5*), III (*CYTB*), IV (*COX1-3*), and V (*ATP6* and 8). Upon transfection in cells for 24 hours, all chimeras blocked translation (Fig. 2D). Moreover, multiplexing could be achieved through transfection of combinations of two chimeras, creating a specific translational block of the targeted mRNAs (Fig. 2E). The specificity of chimera-mediated silencing in living cells was further supported by their selective effects on mRNA-ribosome association. Targeting the 5’ region of *COX1, COX2*, and *CYTB* mRNAs led to a specific reduction of association of the cognate mRNA with the mitochondrial ribosome (Fig. 2F).

The demands on mitochondrial oxidative phosphorylation differ significantly between cell types, and questions regarding mitochondrial gene expression must be assessed in metabolically-specialized cellular contexts. Hence, we transfected chimeras into human iPS cell-derived cardiomyocytes and mouse liver cells (AML12 line). All tested chimeras were efficiently transfected into the cells and blocked translation of the cognate mRNAs (Fig. 2, G and H).

Thus, transfecting presequence-morpholino chimeras into cells can block the translation of mitochondrial mRNAs in different biological contexts.

## Transcriptional responses to chimera-mediated silencing

**Fig. 3:**
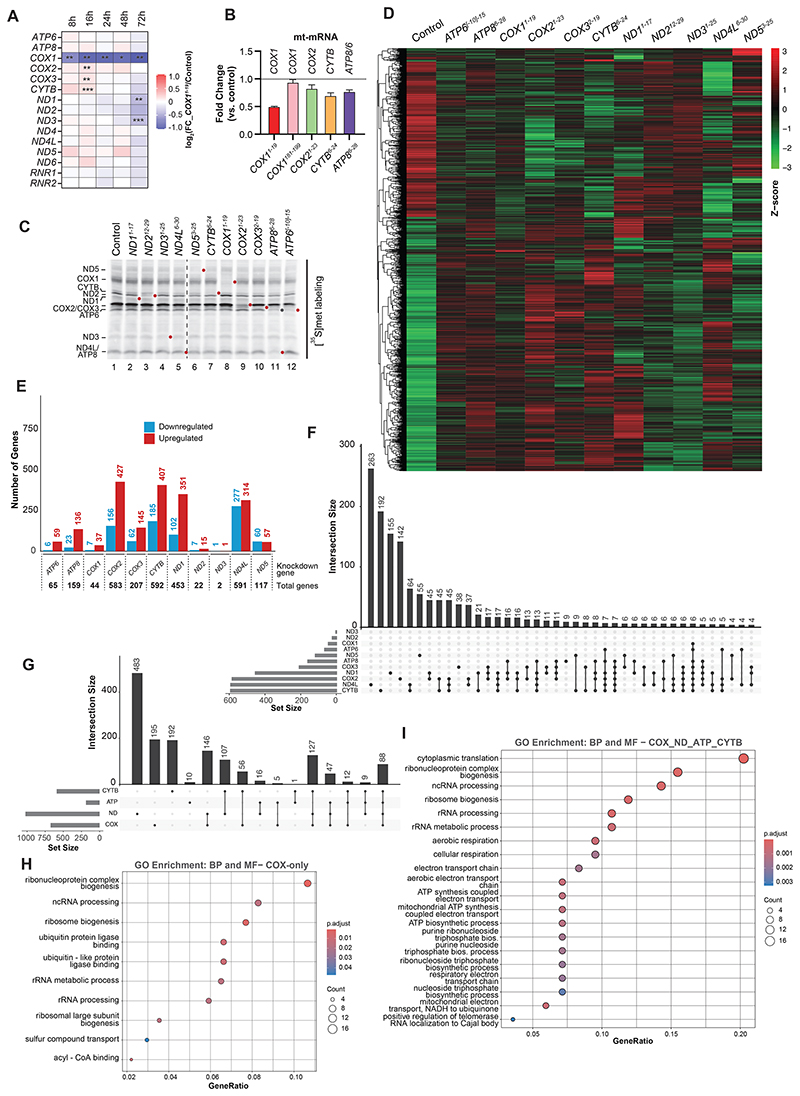
Silencing of mtDNA-encoded genes in living cells alters RNA abundance. See also fig. S3, S4, and S5. See also table S1 and S2. (**A**) Heatmap representing the abundance of mtDNA-encoded mRNAs and rRNAs upon treatment with pCox4-*COX1*^*1-19*^ compared to control (pCox4) as determined by nanoString. FC, fold change (mean, n=4). Statistical significance was determined by multiple t test using the Holm-Sidak method, with alpha=0.05 (^*^, padj<0.05; ^**^, padj<0.01; ^***^, padj<0.001). (**B**) Effect of indicated chimera treatment on cognate mRNA abundance (mean ± SEM, n=3), compared to control (pCox4)(dashed line). (**C**) Mitochondrial translation silenced in cells for 48 hours for transcriptomics’ sample preparation. Newly-synthesized proteins were [^35^S]methionine labelled. Targeted protein indicated by red dot; ATP6 upon ATP8 silencing indicated by gray dot. (**D**) Heatmap of clustering for significant variation of mRNA abundances upon 48 hours silencing. 1503 genes with significantly changed (padj<0.05) mRNA abundance under at least one condition (as in E) are shown. Total RNA was extracted from cells, mRNA enriched, and analyzed by RNA-seq. (**E**) Number of genes significantly changed (padj<0.05) upon treatment for each condition compared to the control (pCox4). The amount of downregulated (blue), upregulated (red), and total genes (at the bottom) are indicated for each gene silenced. (**F**) Data of altered mRNA abundance through UpSet visualization. Number of genes in each exclusive intersection is indicated for each subset as intersection size. The set size (same as total genes in E) is also represented. (**G**) Total genes significantly changed as represented in (E) were first grouped according to the related OXPHOS complexes into ND (ND1∪2∪3∪4L∪5), CYTB, COX (COX1∪2∪3), and ATP (ATP6∪8), and then represented by the UpSet algorithm. The size of each exclusive intersection is indicated, as well as the set size. (**H**) Gene functional enrichment analysis by gene ontology annotation of mRNAs that significantly varied in all groups (ND_CYTB_COX_ATP intersection) (**I**) Gene functional enrichment analysis by gene ontology annotation of mRNAs that significantly varied only upon silencing of complex IV subunits (COX).

Silencing of mitochondrial translation in purified mitochondria does not impact mitochondrial mRNA levels (19). To address if this was also true under silencing conditions in living cells, we monitored mitochondrial mRNA levels upon COX1 silencing by nanoString technology over 72 hours. We noted that *COX1* transcript levels dropped rapidly upon treatment with the chimera. In contrast, other transcripts were not significantly affected in the first 48 hours but started to show a slight reduction upon 72 hours of silencing (Fig. 3A). Of the tested transcripts and chimeras, the *COX1* mRNA was strongly reduced in the presence of the cognate chimera (Fig. 3B).The treatment with a chimera that targets an internal region within the *COX1* mRNA (*COX1*^*181-199*^) did not lead to a reduction in transcript levels (Fig. 3B). Moreover, translation inhibition by thiamphenicol led to a decrease in *COX1* transcript abundance. This effect was exacerbated by treatment with *COX1*^*1-19*^ chimera (fig. S2G). Hence, the reduction of *COX1* mRNA abundance was due to COX1 translation impairment rather than the formation of a morpholino-RNA hybrid. The *COX1* mRNA is the most abundant mitochondrial transcript (21) and these findings suggest that it is apparently prone to specific quality control processes.

A major goal of silencing mitochondrial gene expression in living cells was to address the integration of mitochondrial translation into cellular physiology. We hypothesized that the transcriptional program of the nuclear genome would be altered upon silencing of mtDNA-encoded genes. We thus silenced expression of the mtDNA-encoded subunits from complex I (ND1, ND2, ND3, ND4L, ND5), complex III (CYTB), complex IV (COX1, COX2, COX3), and complex V (ATP6, ATP8) for 48 hours and monitored alterations in gene expression by mRNA sequencing (RNA-seq) (Fig. 3C and Table S1). Distinct changes were observed in the mRNA abundance patterns among the different treatments (Fig. 3D). Moreover, silencing of certain genes led to broader cellular responses as in the case of ND1, ND4L, CYTB, and COX2 (Fig. 3E). To further illustrate the similarities and differences among the treatments, we determined the exclusive intersection subsets considering all significantly-altered transcripts in each condition and using the UpSet visualization approach (22)(Fig. 3F). Here, the term “exclusive” refers to elements that are not shared with other subsets (equivalent to the atomic areas of a Venn diagram). At first glance, in treatments targeting ND1, ND4L, ND5, CYTB, COX2, or COX3, the major intersection size was the subset of transcripts specifically altered in their corresponding sets (i.e., ND1-, ND4L-, ND5-, CYTB-, COX2-, and COX3-only, respectively). Nevertheless, we also observed common responses among the treatments, as indicated by the size of exclusive intersections (e.g., ND1_COX2, COX2_CYTB) (Fig. 3F and Table S2).

To reduce data dimensionality and focus on global responses, we grouped the mRNAs altered upon silencing of subunits of OXPHOS complexes into treatment sets, ND (ND1∪ND2∪ND3∪ND4L∪ND5), CYTB (CYTB), COX (COX1∪COX2∪COX3), and ATP (ATP6∪ATP8). Using these groups, we performed gene ontology enrichment analysis (GO) on the biological process (BP) and molecular function (MF) for each group. We observed a marked representation of cytoplasmic translation, metabolism, energy, and OXPHOS function pathways (fig. 3S, A-D). We then determined the exclusive intersection subsets considering all transcripts in each group (Fig. 3G and Table S2). As in the case of individual treatment data (Fig. 3F), the most represented subsets were those transcripts specific for each condition: ND-, COX-, and CYTB-only subsets include 483, 195, and 192 genes, respectively (Fig. 3G). Although GO analyses showed that similar pathways were enriched for COX- and ND-only subsets, the genes involved differed. We also observed the enrichment of characteristic pathways for each COX- and ND-only subsets (Fig. 3H and fig. S3E, S4A).

When we determined the exclusive interception of the ND, CYTB, COX, and ATP groups, we obtained a subset of 88 transcripts that changed in all four groups (Fig. 3G). The protein products of these mRNAs were involved in gene expression (mainly cytosolic and mitochondrial translation) and cellular respiration (Fig. 3I and fig. S4B). Additionally, 127 transcripts specifically changed in the groups corresponding to the electron transport chain complexes (ND_CYTB_COX subset) (Fig. 3G). These genes were mainly involved in metabolism-related pathways, gene expression, organelle organization, and OXPHOS function (fig. S5).

Thus, mitochondria-nucleus communication displays a specific pattern with regard to the targeted mitochondrial mRNA and the response of nuclear gene expression appears to be more hierarchical than previously suggested (23–25). The ablation of subunits forming different OXPHOS complexes (e.g., COX2 vs. CYTB) or of different core components in the same complex (e.g., COX1 vs. COX2 vs. COX3) had disparate influence on the cellular gene expression profile.

## Selective alterations in mitochondrial function upon gene silencing

**Fig. 4:**
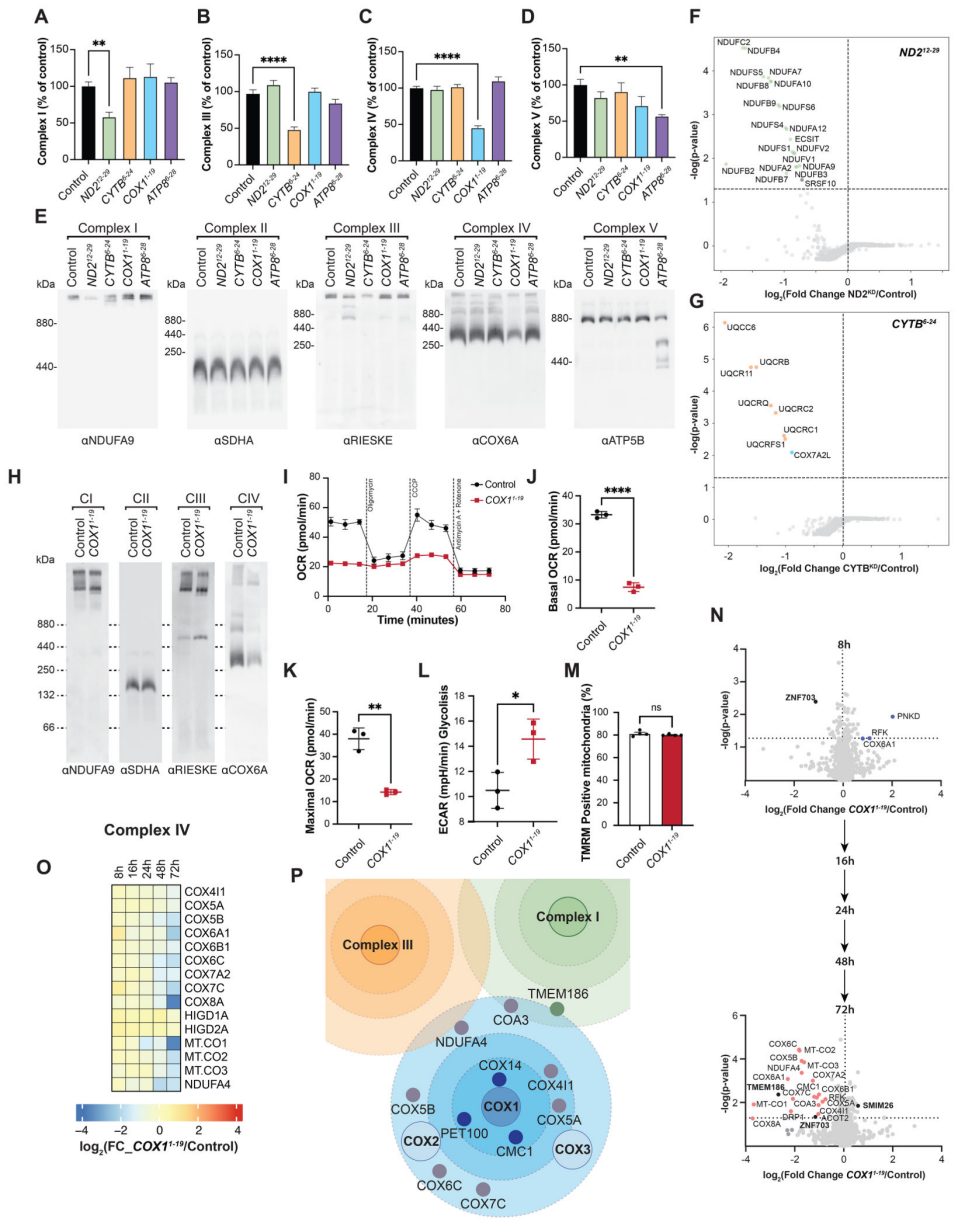
Proteome remodeling of mitochondria upon silencing in living cells. See also fig. S6. See also table S3 and S4. (**A-D**) Mitochondrial OXPHOS complex activity after *ND2, CYTB, COX1*, and *ATP8/ATP6* mRNA silencing in HEK293T cells for 48 hours. Represented as percentage of the control (n=4, mean ± SEM). Statistical significance was determined by unpaired t test (^**^, p<0.01 and ^****^, p<0.0001). (**E**) Immunodetection of mitochondrial OXPHOS complexes analyzed by BN-PAGE after treatment of HEK293T cells with chimeras silencing ND2, CYTB, COX1, and ATP8 for 48 hours. **(F-G)** Proteomic analyses of mitochondria isolated from cells treated with *ND2*^*12-29*^ (F) and *CYTB*^*6-24*^ (G) chimeras for 48 hours (n=4). Rank sum plots display variation in protein abundance between silenced cells (*ND2*^*12-29*^ and *CYTB*^*6-24*^), represented as fold change compared to the control. Proteins associated to assembly of complexes I, III, and IV indicated in green, orange, and blue, respectively. (**H**) Immunodetection of OXPHOS mitochondrial complexes by BN-PAGE after treatment of HEK293T cells with *COX1*^*1-19*^ for 72 hours. CI, II, III, IV, & V: complex I, II, III, IV, & V, respectively. (**I-K**) Real time respirometry of HEK293T cells treated with pCox4-*COX1*^*1-19*^ for 72 hours. The basal (**J**) and maximal (**K**) oxygen consumption rates (OCR) were significantly decreased compared to the control treatment (pCox4) (^****^, p<0.0001 and ^**^, p<0.01) (n=3, mean ± SEM). (**L**) Extracellular acidification rate (ECAR*)* in cells treated with pCox4-*COX1*^*1-19*^ for 72 hours compared to the control (pCox4)(^*^,p<0.05) (n=3, mean±SEM). (**M**) Mitochondrial membrane potential measured by TMRM in HEK293T cells treated with pCox4-*COX1*^*1-19*^ for 72 hours (ns, not significant) (n=4, mean ± SEM). Statistical significance in (J-M) was determined by unpaired t test. (**N**) Effect of COX1 knockdown on the mitochondrial proteome analyzed by quantitative MS analyses of mitochondria from HEK293T cells treated with *COX1*^*1-19*^ for 8, 16, 24, 48, and 72 hours. **(O)** Heatmap of quantitative MS analyses of complex IV subunits. (**P**) Diagram illustrating gradual and sequential propagation of OXPHOS defects upon COX1 silencing.

To assess the consequences of gene silencing at the organelle level, we transfected chimeras in HEK293T cells, blocking the translation of ND2 (complex I), CYTB (complex III), COX1 (complex IV), and ATP8 (complex V). After 48 hours of treatment, we monitored OXPHOS complex activities. For ND2, CYTB, COX1, and ATP8 silencing, we observed a robust and selective decrease in the activity of the corresponding complex (I, III, IV, and V, respectively) (Fig. 4, A-D). As shown by Blue Native (BN) polyacrylamide gel electrophoresis (PAGE), ND2 and CYTB silencing led to decreased levels of complex I and III, respectively, and altered CI-CIII supercomplex assembly state (Fig. 4E). COX1 silencing caused a decrease of complex IV, while ATP8 silencing (downregulating ATP8 and ATP6, subunits of the F_o_ domain) led to reduction of complex V and appearance of free F_1_ domain (Fig. 4E). Complex II levels remained unaffected in all conditions (Fig. 4E). These findings were further supported by proteomic analysis of mitochondria from HEK293T cells treated with ND2 and CYTB chimeras for 48 hours (Table S3). Significant and specific reduction of protein subunits corresponding to complex I (in particular ND2-, N-, and ND5-module) (Fig. 4F) and complex III (Fig. 4G) were observed upon ND2 and CYTB silencing, respectively (26).

As judged by mitochondrial translation experiments, extended COX1 silencing did not indirectly affect the synthesis of other mitochondrial-encoded proteins (Fig. 2A). Upon treatment with pCox4-*COX1*^*1-19*^ chimera for 72 hours, we observed a drastic and selective loss of complex IV (Fig. 4H). While the abundance of other tested complexes remained unaffected, the loss of complex IV led to reduced supercomplex formation apparent in an altered complex profile of complexes I and III, more evident than after 48 hours of treatment (Fig. 4H). Correspondingly, real-time respirometry measurements revealed a significant reduction of O_2_ consumption rates in the chimera-treated samples (Fig. 4, I-K). The loss of OXPHOS capacity led to a metabolic shift of the treated cells to glycolytic metabolism (Fig. 4L, fig. S6, A and B). However, the mitochondrial membrane potential was still maintained under these conditions (Fig. 4M).

Because COX1 silencing was effective from three to 72 hours, we performed time-resolved proteomic analyses to assess the effect unbiasedly. To this end, we transfected the pCox4-*COX1*^*1-19*^ chimera to silence COX1 expression in HEK293T cells for 8, 16, 24, 48, and 72 hours (fig. S6C). Quantitative mass spectrometric analyses of the isolated mitochondria were carried out to determine changes in the mitochondrial proteome (Table S4) (27). At 8 hours, only a few mitochondrial proteins were altered in their abundance. The largest changes were observed for ZNF703 and PNKD (Fig. 4N). Extended silencing increased the number of mitochondrial proteins with altered abundance (Fig. 4N). The amount of complex IV subunits gradually decreased over time, while other OXPHOS complexes were not affected (Fig. 4O and fig. S6D). In addition, biogenesis factors for complex IV (COX14, COA3, CMC1, PET100) (26, 28) displayed reduced levels upon COX1 silencing. Because we observed reduced levels of DRP1 (Fig. 4N) (29, 30), we assessed mitochondrial network organization by microscopy but found that it was unaffected (fig. S6E).

In summary, silencing of mitochondrial gene expression led to highly specific alterations of the mitochondrial proteome that could be monitored over a time period from hours to days. In this context, the loss of complex I, III, or IV (upon silencing of ND2, CYTB, and COX1, respectively) did not substantially alter the abundance of other OXPHOS complexes or their subunits. Our results revealed the sequence of events in which the ablation of COX1 first affected COX1-module proteins, complex IV assembly factors, COX2 and COX3-modules, and was later transmitted to other OXPHOS complexes through dual function assembly factors (COA3, TMEM186, etc.) (Fig. 4P).

## Identification of mitochondrial biogenesis factors

To address their functional significance, we selected three proteins from the proteomic data - ZNF703, TMEM186, and SMIM26 - that displayed altered abundance (Fig. 4N) and that are reported to localize to mitochondria (27). Proteinase K protection and mitochondria sub-fractionation confirmed TMEM186 as an integral inner membrane protein (fig. S7, A-B) (31). Although a considerable fraction of ZNF703 was protease accessible in intact mitochondria and thus likely associated with the outer mitochondrial membrane (OMM), a fraction was protected by the inner membrane, and ZNF703 appeared partially membrane-associated (fig. S7, C-D). SMIM26 was located in the inner mitochondrial membrane (IMM) and was sensitive to protease treatment in mitoplasts (fig. S7, E-F).

**Fig. 5:**
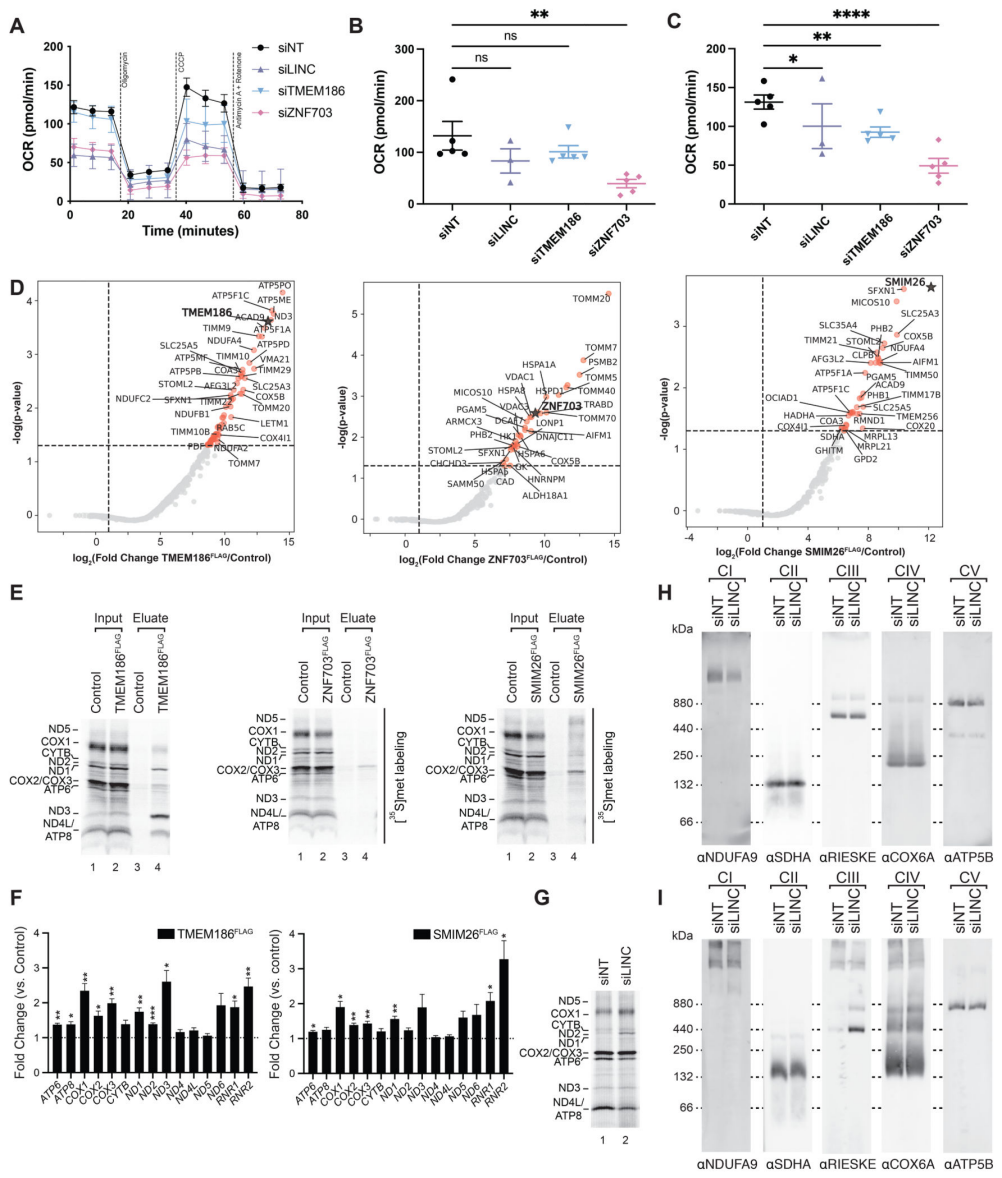
Identification of mitochondrial biogenesis factors. See also fig. S7. See also table S5. **(A-C)** Real time respirometry measurements of HEK293T after siRNA-mediated downregulation of TMEM186 (siTMEM186), ZNF703 (siZNF703), and *LINC00493* (siLINC). Control, cells treated with non-targeting siRNA (siNT). Representative profile of oxygen consumption rate (OCR) (**A**), basal OCR (**B**) (mean ± SEM; n=5 for all except siLINC where n=3), and maximal OCR (**C**) (mean ± SEM; n=5 for all except siLINC where n=3). Statistical significance was determined by 2-way ANOVA with multiple comparisons (ns, not significant; ^*^, p<0.05; ^**^, p<0.01; ^****^, p<0.0001). (**D**) Quantitative mass spectrometry analyses to determine TMEM186, ZNF703, and SMIM26 interaction partners. TMEM186^FLAG^, ZNF703^FLAG^, and SMIM26^FLAG^ were expressed in HEK293T cells, mitochondria isolated, solubilized, and subjected to FLAG-IP. Eluates (including the control) were analyzed by label-free mass spectrometry and protein enrichment represented as log_2_ (Fold Change) (n=4). Significantly enriched proteins are indicated in orange. The bait is presented in bold with a star in each case. (**E**) TMEM186^FLAG^, ZNF703^FLAG^, and SMIM26^FLAG^ co-precipitated newly synthesized mtDNA-encoded proteins. Total, 3%; eluate 100%. (**F**) Determination of mitochondrial mRNAs and rRNAs co-precipitating with TMEM186^FLAG^ and SMIM26^FLAG^ by nanoString technology. Results are presented as fold change to the Control (WT cells, dashed line) (mean ± SEM, n=3). Statistical significance was determined by multiple t test using the Holm-Sidak method, with alpha=0.05 (^*^, padj<0.05; ^**^, padj<0.01; ^***^, padj<0.001). (**G**) Mitochondrial translation products were labeled with [^35^S]methionine after siRNA-mediated downregulation of *LINC00493* (siLINC) in HEK293T cells. Control, non-targeting siRNA (siNT). (**H-I**) Immunodetection of mitochondrial respiratory complexes by BN-PAGE after siRNA-mediated downregulation of *LINC00493* (siLINC) in HEK293T cells. Isolated mitochondria were solubilized with 1% DDM (**H**) or Digitonin (**I**). CI, complex I; CII, complex II; CIII, complex III; CIV, complex IV; CV, complex V.

siRNA-mediated knockdown of each of the three proteins reduced mitochondrial OXPHOS capacity, supporting a functional link to mitochondrial biology (Fig. 5, A-C). To elucidate their role in mitochondria, we defined interaction partners by immunoisolation of FLAG-tagged protein versions and mass spectrometric analysis (Fig. 5D and Table S5).

TMEM186 is implicated in early steps of complex I biogenesis (31, 32). Accordingly, TMEM186^FLAG^ co-isolated complex I subunits (ND3, NDUFC2, NDUFB1) but also proteins involved in complex IV biogenesis (COX5B, COA3, COX4-1), complex V, and the TIM22 complex (Fig. 5D). In addition, TMEM186^FLAG^ immunoisolation after [^35^S]methionine labeling of mitochondrial translation products co-isolated newly-synthesized ND2, ND3, and ND4L. We also recovered COX1, COX2, and COX3 (Fig. 5E) in the eluate together with the corresponding mRNAs and rRNAs (Fig. 5F). 2D BN-/SDS-PAGE analysis revealed the association of TMEM186 with assembly intermediates of the newly-synthesized mitochondrial-encoded proteins (fig. S7G). These findings extended the role of TMEM186 from mitochondrial complex I to complex IV biogenesis.

The zinc-finger protein 703 (ZNF703) is an oncogene in different types of cancer (33, 34) that localizes to the nucleus (35) and mitochondria (27). ZNF703^FLAG^ interacted with the translocase of the outer mitochondrial membrane (TOM complex), the sorting and assembly machinery (SAM complex), and the voltage-dependent anion channel (VDAC). Moreover, it co-purified proteins of the inner membrane, including MICOS (mitochondrial contact site and cristae organizing system), the apoptosis-inducing factor 1 (AIFM1), and the COX2-module subunit COX5B (Fig. 5D). We assessed whether ZNF703 participated in OXPHOS complex biogenesis. ZNF703^FLAG^ immunoisolation after [^35^S]methionine labeling specifically co-isolated newly-synthesized COX2 (Fig. 5E) as part of a high-molecular-weight complex (fig. S6G). This suggested that ZNF703 supports COX2 biogenesis in the context of the MICOS network.

The *LINC00493* gene encodes the long non-coding RNA *LINC00493* and the small mitochondrial membrane protein SMIM26 (fig. S7I) (36, 37). SMIM26 levels were increased upon COX1 silencing for 72 hours (Fig. 4O). Among SMIM26^FLAG^ interactors, we identified several proteins required for complex IV biogenesis (COA3, COX4I1, COX20, and COX5B), quality-control at the IMM (AFG3L2, PHB1, PHB2, CLPB), and the mitochondrial ribosome (Fig. 5D and fig. S7K).

We assessed whether SMIM26 was an early assembly factor for mtDNA-encoded proteins by SMIM26^FLAG^ immunoisolation after [^35^S]methionine metabolic labeling of mitochondrial translation products. SMIM26 interacted with newly synthesized COX1, COX2, and ND5 (Fig. 5E and fig. S7J). The fuzzy background in the eluate suggested that SMIM26 interacted with nascent chains (Fig. 5E). In agreement with this, SMIM26 immunoisolation showed co-isolation of mitochondrial rRNAs and mRNAs encoding complex I and IV subunits (Fig. 5F). [^35^S]methionine labeling of mitochondrial translation products in siRNA-mediated *LINC00493/SMIM26* knockdown in HEK293T cells resulted in increased synthesis of ND2 and COX1 (Fig. 5I). However, depletion of *LINC00493/SMIM26* resulted in reduced levels of complex I (Fig. 5H), concomitant with a reduction of supercomplexes that led to increased amounts of free complex III dimer (Fig. 5I). Nevertheless, the abundance of mitochondrial mRNAs and rRNAs was not markedly affected upon TMEM186, ZNF703, and *LINC00493/SMIM26* ablation through siRNA-mediated knockdown (Fig. S7L).

In summary, mitochondrial proteome alterations observed in the context of COX1 silencing allowed us to identify proteins linked to mitochondrial gene expression processes and, concomitantly, OXPHOS assembly and function.

## Conclusion

Here we developed an experimental strategy that enabled the robust and specific silencing of mitochondrial mRNA expression in living cells. By using presequence-morpholino chimeras generated by click chemistry, we specifically blocked the translation of transcripts within a timeframe of three to 72 hours. This approach allowed us to investigate the effects of silencing mtDNA-encoded mRNAs on mitochondrial proteomes and nuclear gene expression. Our results revealed complex interactions between mitochondria and the nucleus and provided insights into the regulation of mitochondrial biogenesis and gene expression. We also identified factors that impact mitochondrial biogenesis and cellular homeostasis, including TMEM186, ZNF703, and SMIM26. Our findings challenge the assumption that the loss of complexes I, III, and IV of the OXPHOS system would have an immediate cascading effect on other complexes in the system. The availability of this chimera-based mitochondrial mRNA silencing strategy provides a valuable tool for researchers to study mitochondrial gene expression, protein biogenesis, and their integration into cellular physiology.

## Materials and methods summary

### Synthesis of Jac1- and peptide-morpholino oligonucleotide (MO) chimera

Jac1-MO chimeras were synthesized as previously described (19). Briefly, Jac1-DBCO (Dibenzocyclooctin) was mixed with an 8-fold molar excess of morpholino phosphorodiamidate oligonucleotides (MO) (Gene Tools) containing 5’-[azide] and 3’-[FITC] groups. The click reaction was carried out at 25°C for two hours with constant mixing. Peptide-MO chimeras were synthesized by a click chemistry reaction. pCox4^1-25^ (*Saccharomyces cerevisiae* Cox4 presequence), containing a lysine-azide group at the C-terminus (MLSLRQSIRFFKPATRTLCSSRYLL{Lys(N_3_)}-amide) (GenScript) was mixed in a molar ratio of 1:1 with morpholino phosphorodiamidate oligonucleotides (MO) (Gene Tools) containing 5’-[cyclooctyne] and 3’-[FITC] groups, final concentration of 100 μM. The click reaction was carried out at 20°C overnight, with periodic mixing (cycles of 30 sec mixing, 30 min rest). Chimeras used for transfection in cell culture were sterile-filtered with small 0.22 μm filters. The morpholinos used in this study with the cyclooctyne group are described in the Supplementary material. The morpholino used to prepare the chimera Jac1-COX1^1-19^ contained 5’-[azide] and 3’-[FITC] groups as previously (19).

### mtDNA-encoded gene silencing in living cells

HEK293T, AML12, and HeLa cells were seeded in 12-well cell culture plates (80,000 cells/well), 6-well plates (16,0000 cells/well), and T25 cell culture flask (400,000 cells/flask). The cells were incubated for 24 hours at 37°C with 5% CO_2_. For 72 hours silencing treatment, the media was exchanged to fresh media containing Endo-Porter (GeneTools) at 2 μM final concentration. Unless indicated otherwise, chimeras were added at 2 μM final concentration. For 24 and 48 hours treatments, the media was initially exchanged to fresh media. After 24 or 48 hours, the media was one more time exchanged, but to fresh media containing 2 μM Endo-Porter and 2 μM chimera to start the silencing, and incubated for 48 and 24 hours, respectively. The cells were then harvested or used for further analyses. Human iPS cell-derived cardiomyocytes (obtained from the Stem Cell unit at University Medical Center Goettingen) were seeded at a cell density of approximately one million cells per well in a 6-well plate. Media was exchanged to fresh media containing 4 μM Endo-Porter and 2.5 μM chimera, and incubated for 24 hours. The cells were then harvested or used for further analyses. The chimera and Endo-Porter concentrations have to be titrated for each cell type to guarantee efficient, robust, and reproducible mtDNA gene silencing.

### siRNA-mediated protein knockdown

siRNAs targeting ZNF703, TMEM186, and *LINC00493* were purchased from Horizon Discovery (UK). HEK293T cells (1x10^6^ cells) were transfected with Lipofectamine RNAiMAX (Invitrogen) following the manufacturer’s instructions and transferred to a T25 cell culture flask. Cells were transfected for 72 hours and then used for further analyses. In experiments requiring mitochondria isolation, the transfection mix was scaled-up accordingly. siRNA was used at 33 nM final concentration in all experiments.

### Transient expression of FLAG-tagged proteins

The open reading frame (ORF) of TMEM186 (NM_015421.4), ZNF703 (NM_025069.3), and SMIM26 (NM_001348957.2) inserted in pcDNA3.1 plasmid, in frame with a C-terminal FLAG tag, were purchased from GenScript (NL). For FLAG immunoisolation experiments, plasmids were transfected using PEI (polyethylenimine) transfection reagent (Polysciences Europe). Briefly, HEK293T cells were seeded to a confluency of 50% two days prior to transfection in a 145 mm cell culture dish. 100 µL PEI (stock 10 mg/ml) were mixed with 400 µL OptiMEM, incubated at RT for 5minutes, and 4.5 mL of OptiMEM were added. For the transfection, 1 mL PEI/OptiMEM mix was transferred to a new tube containing 20 µg plasmid DNA in 1 mL OptiMEM, followed by mixing. After a 20 minutes incubation at RT, 10 mL of DMEM media supplemented with 10% (v/v) dialyzed fetal bovine serum, 2 mM L-glutamine, 1 mM sodium pyruvate, and 50 μg/mL uridine was added. The mixture was carefully transferred to the cell culture dish. After one hour incubation under standard growth condition, 10 mL of fresh media was added to the cells, and further incubated for 24 hours prior harvest.

### Mitochondrial RNA detection by nanoString technology

Immunoisolation input and eluate fractions were mixed with Trizol reagent (Thermo Scientific Fisher) and the RNA purified using RNA Clean & Concentrator kit (Zymo Research) following the manufacturer’s instructions. For the analysis of siRNA-treated cells, mitochondria were isolated and solubilized in immunoprecipitation’s lysis buffer with 1% digitonin as described above. The cleared lysate was then processed for RNA isolation. Equivalent amounts of RNA were mixed with a TagSet-24 and detection primers (IDT) previously used to detect mitochondrial transcripts (19, 41). Next, the samples were processed and analyzed in a nCounter® MAX analysis system (nanoString) following the manufacturer’s instructions. The acquired data were analyzed with nSolver software (nanoString).

### RNA sequencing and analysis

HEK293T cells were treated for 48 hours with chimeras targeting different mtDNA-encoded mRNAs. Control samples were transfected with pCox4 in the click reaction buffer. After discarding the media, cells were resuspended in Trizol reagent. The RNA was purified using RNA Clean & Concentrator kit (Zymo Research) following the manufacturer’s instructions. RNA quality control was assessed in a Fragment Analyzer. The NovaSeq X Plus sequencing platform (Illumina, USA) was used to perform 50 bp paired-end sequencing on the samples with 9 G raw data per sample. For each treatment, four biological replicates were analyzed, except for COX2 and ND2 where three biological replicates prepared and measured.

The downstream analysis was performed in RStudio (R version 4.3.0) using packages from the Bioconductor repository (42, 43) and the Tidyverse suite.

Differential gene expression analysis was conducted using DESeq2 (version 1.40.2) (44). Batch correction was applied using limma (version 3.56.2) (45) to account for variability between replicates performed on different days with different reagent batches (batch1: replicate 1 and 2; batch 2: replicate 3 and 4) to improve clustering and reliability of differential expression results.

For Gene Ontology (GO) term enrichment analysis, the clusterProfiler (version 4.8.3) (46) package was used, with annotations from org.Hs.eg.db (version 3.17.0), focusing on Biological Processes (BP) and Molecular Functions (MF). UpSet plots were generated using UpSetR (version 1.4.0) (47) to visualize intersections of significantly changed genes across conditions. Graphical representations were generated through the ggplot2 package (version 3.5.1).

### Measurement of mitochondrial complex I, III, IV and V activities in HEK293T cells

HEK293T cells were transfected with chimeras for 48 hours. Mitochondrial respiratory chain complex activities were measured using enzyme activity microplate assay kits from Abcam: Complex I Enzyme Activity Microplate Assay Kit (ab109721), Mitochondrial Complex III Activity Assay Kit (ab287844), Complex IV Rodent Enzyme Activity Microplate Assay Kit (ab109911), and ATP Synthase (Complex V) Enzyme Activity Microplate Assay Kit (ab109714). The assays were performed according to the manufacturer’s instructions, using HEK293T cell lysates for complexes I, IV, and V, and isolated mitochondria from HEK293T cells for complex III. All samples were kept on ice throughout processing. For complex I measurements, 100 µg protein of cell lysate were loaded per well in a 96-well plate; for complex III, 5 µg isolated mitochondria were used per reaction; in case of complex IV, 20 µg protein of cell lysate were used; for complex V, 50 µg protein of cell lysate were used per reaction. All absorbance measurements were conducted using a Synergy H1 microplate reader (BioTek). Enzymatic activities were calculated by determining the rate of absorbance change over time.

### Quantification and statistical analysis

Autoradiographic and immuno blot signal intensities were quantified with ImageQuantTL v8.1 (GE Healthcare) and ImageJ v1.47 (NIH). Data were obtained from three or more biological replicates (n), and were processed with GraphPad Prism 8 software for statistical purposes. Mean, SEM, and statistical significance are listed in figure legends.

An extended description of the Materials and methods in included in the Supplementary Materials.

## Supplementary Material

Supplementary Material

## Data Availability

Requests for reagents should be directed to and will be fulfilled by the lead author. RNA-seq data was deposited to the GEO database under the accession number GSE292101 (https://www.ncbi.nlm.nih.gov/geo/query/acc.cgi?acc=GSE292101). The custom R code used to operate RStudio for the transcriptomic data analysis is available from Zenodo (https://doi.org/10.5281/zenodo.15260712). The mass spectrometric data have been deposited to the ProteomeXchange Consortium via the PRIDE (38) partner repository with the dataset identifiers PXD061846 (CYTB^KD^ and ND2^KD^ data) (https://www.ebi.ac.uk/pride/archive/projects/PXD061846), PXD061876 (COX1^KD^ data) (https://www.ebi.ac.uk/pride/archive/projects/PXD061876), and PXD061877 (FLAG IP data) (https://www.ebi.ac.uk/pride/archive/projects/PXD061877). The Python code used to operate the autoprot package for the mass spectrometric data analysis is available from Zenodo (https://doi.org/10.5281/zenodo.15241939).
