# Supplementary material for: Silencing mitochondrial gene expression in living cells
Source: Science. Author manuscript; Available in PMC 2025 Oct 18. (PMC7618265; doi:10.1126/science.adr3498)
Supplement: Supplementary Material [file EMS209270-supplement-Supplementary_Material.zip › science.adr3498_mdar_reproducibility_checklist.pdf]

Article Metadata

**Title**	Silencing mitochondrial gene expression in living cells
**Publication URL**	https://doi.org/10.1126/science.adr3498
**Data Availability Statement**	Data and code availability<br>▪ This paper does not report original code.<br>▪ Any additional information required to reanalyze the data reported in this paper is available from the lead contact upon request.
**AAAS survey (please complete)**	https://airtable.com/appEyo44c4qrc3VQE/shrOyR475L66qpU3v

Materials:

Newly created materials	Materials Availability Statement	Article Subsection	DataSeer Notes	Author Response
The manuscript includes a dedicated "materials availability statement" providing transparent disclosure about availability of newly created materials including details on how materials can be accessed and describing any restrictions on access.	Materials availability<br>Plasmids, cell lines, and other resources are available upon request to the lead contact.		Statement included in article text.	

Antibodies

No Action Required | *Optional*

Material is not required, but is recommended to be registered for an identifier to be included in the article text.

Name	Associated sentence from text	Article Subsection	Source	Identifier	DataSeer Notes	Author Response
anti-TOM20	Following a washing step with PBS, the primary antibody solu	Immunofluorescence staining,	Abcam		Listed in article text	
secondary anti-rabbit antibody from goat	After three washing steps with PBS, the secondary antibody s	Immunofluorescence staining,	AffiniPure		Listed in article text	

DNA and RNA Sequences

No Action Required | *Done - Shared/Cited*

Material is sufficiently shared/cited

Name	Associated sentence from text	Article Subsection	Source	Identifier	DataSeer Notes	Author Response
ND1 1-17	The morpholinos used in this study were: ND1 1-17 , AGGTT	Synthesis of Jac1-and peptide		AGGTTGGCCATGGGTA	Sequence shared in article text	
ND2 12-29	The morpholinos used in this study were: ND1 1-17 , AGGTT	Synthesis of Jac1-and peptide		TAGATGACGGGTTGGG	Sequence shared in article text	
ND3 1-25	The morpholinos used in this study were: ND1 1-17 , AGGTT	Synthesis of Jac1-and peptide		TTATTAAAATTAAGGCG	Sequence shared in article text	
ND4L 6-30	The morpholinos used in this study were: ND1 1-17 , AGGTT	Synthesis of Jac1-and peptide		TAGTATAATATTTATGTA	Sequence shared in article text	
ND4 [-15]-10	The morpholinos used in this study were: ND1 1-17 , AGGTT	Synthesis of Jac1-and peptide		GTTTTAGCATTGGAGTA	Sequence shared in article text	
ND5 3-25	The morpholinos used in this study were: ND1 1-17 , AGGTT	Synthesis of Jac1-and peptide		TGGTTATAGTAGTGTGC	Sequence shared in article text	
ND6 4-28	The morpholinos used in this study were: ND1 1-17 , AGGTT	Synthesis of Jac1-and peptide		CACTCAACAGAAACAAA	Sequence shared in article text	
CYTB 6-24	The morpholinos used in this study were: ND1 1-17 , AGGTT	Synthesis of Jac1-and peptide		GTTAGTTTTGCGTATTG	Sequence shared in article text	
COX1 1-19	The morpholinos used in this study were: ND1 1-17 , AGGTT	Synthesis of Jac1-and peptide		GTCAACGGTCGGCGAA	Sequence shared in article text	
COX1 181-199	The morpholinos used in this study were: ND1 1-17 , AGGTT	Synthesis of Jac1-and peptide		AGATTATTACAAATGCA	Sequence shared in article text	
COX2 1-23	The morpholinos used in this study were: ND1 1-17 , AGGTT	Synthesis of Jac1-and peptide		CCTACTTGCGCTGCATC	Sequence shared in article text	
COX3 2-19	The morpholinos used in this study were: ND1 1-17 , AGGTT	Synthesis of Jac1-and peptide		CATGTGATTGGTGGGTC	Sequence shared in article text	
ATP8 6-28	The morpholinos used in this study were: ND1 1-17 , AGGTT	Synthesis of Jac1-and peptide		GCCATACGGTAGTATTT	Sequence shared in article text	
ATP6 [-10]-15	The morpholinos used in this study were: ND1 1-17 , AGGTT	Synthesis of Jac1-and peptide		CAGATTTTCGTTCATTT	Sequence shared in article text	
mCOX1 1-24	The morpholinos used in this study were: ND1 1-17 , AGGTT	Synthesis of Jac1-and peptide		GAATAATCAACGATTAA	Sequence shared in article text	
xCOX1 1-22	The morpholinos used in this study were: ND1 1-17 , AGGTT	Synthesis of Jac1-and peptide		ATAATCAACGAGTAATT	Sequence shared in article text	
COX1 19-42	The morpholinos used in this study were: ND1 1-17 , AGGTT	Synthesis of Jac1-and peptide		GTCTTTGTGGTTTGTAG	Sequence shared in article text	
COX1 40-64	The morpholinos used in this study were: ND1 1-17 , AGGTT	Synthesis of Jac1-and peptide		ATAATAGGTATAGTGTT	Sequence shared in article text	
COX1 65-82	The morpholinos used in this study were: ND1 1-17 , AGGTT	Synthesis of Jac1-and peptide		CTCCAGCTCATGCGCC	Sequence shared in article text	
COX1 85-104	The morpholinos used in this study were: ND1 1-17 , AGGTT	Synthesis of Jac1-and peptide		AGGCTTAGAGCTGTGC	Sequence shared in article text	

No Action Required	*Optional*					
Material is not required, but is recommended to be registered for an identifier to be included in the article text.						
**Name**	**Associated sentence from text**	**Article Subsection**	**Source**	**Identifier**	**DataSeer Notes**	**Author Response**
pCox4 1-25	pCox4 1-25 (Saccharomyces cerevisiae Cox4 presequence),	Synthesis of Jac1-and peptide			Listed in article text	
siRNA targeting ZNF703	siRNAs targeting ZNF703, TMEM186, and LINC00493 were	siRNA-mediated protein knock	Horizon Discovery (UK)		Listed in article text	
siRNA targeting TMEM186	siRNAs targeting ZNF703, TMEM186, and LINC00493 were	siRNA-mediated protein knock	Horizon Discovery (UK)		Listed in article text	
siRNA targeting LINC00493	siRNAs targeting ZNF703, TMEM186, and LINC00493 were	siRNA-mediated protein knock	Horizon Discovery (UK)		Listed in article text	
pcDNA3.1	The open reading frame (ORF) of TMEM186 (NM_015421.4)	Transient expression of FLAG			Listed in article text	
pCox4-COX1 1-19 chimera (COX1 1-19 c	Mitochondria-enriched fractions prepared from cells treated w	Quantitative mass spectromet			Listed in article text	
pCox4-ND 12-29 (ND2 12-29 or ND2 KD)	Mitochondria-enriched fractions prepared from cells treated w	Quantitative mass spectromet			Listed in article text	
pCox4-CYTB 6- 24 (CYTB 6-24 or CYTB	Mitochondria-enriched fractions prepared from cells treated w	Quantitative mass spectromet			Listed in article text	
pCox4 (Control)	Mitochondria-enriched fractions prepared from cells treated w	Quantitative mass spectromet			Listed in article text	

Cell Materials

**No Action Required**	*Done - Shared/Cited*					
Material is sufficiently shared/cited						
**Name**	**Associated sentence from text**	**Article Subsection**	**Source**	**Identifier**	**DataSeer Notes**	**Author Response**
AML12 (alpha mouse liver 12) cells	AML12 (alpha mouse liver 12) cells (CRL-2254 ™ , ATCC) we	Mammalian cell culture	ATCC	CRL-2254	Listed in article text	

No Action Required	*Optional*					
Material is not required, but is recommended to be registered for an identifier to be included in the article text.						
**Name**	**Associated sentence from text**	**Article Subsection**	**Source**	**Identifier**	**DataSeer Notes**	**Author Response**
HEK293-Flp-In T-Rex	HEK293-Flp-In TM T-Rex TM (HEK293T), HEK293T-derived,	Mammalian cell culture			Listed in article text	
HeLa	HEK293-Flp-In TM T-Rex TM (HEK293T), HEK293T-derived,	Mammalian cell culture			Listed in article text	
Human iPS cell-derived cardiomyocytes	Human iPS cell-derived cardiomyocytes were cultured in RPI	Mammalian cell culture			Listed in article text	
HEK293T mL45FLAG cells	HEK293T mL45FLAG cells (19) were treated with 0.6 mg/mL	Mammalian cell culture			Listed in article text	

Experimental Animals

output type not detected in text

Plants and Microbes

output type not detected in text

Human Research Participants

output type not detected in text

Design:

Study protocol

output type not detected in text

Laboratory protocol

output type not detected in text

Ethics

output type not detected in text

Analysis:

Data availability

**No Action Required**	*Done - Shared/Cited*					
Research outputs are appropriately shared/cited						
**Datatype**	**Associated sentence from text**	**Article Subsection**	**URL**	**Accession/DOI/PID**	**DataSeer Notes**	**Author Response**
Genetic Data: High-Throughput Nucleotide Sequencing	The NovaSeq X Plus sequencing platform (Illumina, USA) was used to perform 50 bp paired-end sequencing on the samples with 9 G raw data per sample.	RNA sequencing and analysis	https://www.ncbi.nlm.nih.gov/geo/query/acc.cgi?acc=GSE292101	GSE292101672	Accession "GSE292101" is currently private and is scheduled to be released on Mar 15, 2026.	
Mass Spectrometry: Liquid Chromatography Mass Spectrometry	Following colloidal Coomassie Blue staining, gel lanes were cut into five pieces each and processed for liquid chromatography-mass spectrometry (LC-MS) analysis as described before (46).	Quantitative mass spectrometry analysis	https://www.ebi.ac.uk/pride/archive/projects/PXD061846	PXD061846	Properly shared on PRIDE	
Mass Spectrometry: Liquid Chromatography Mass Spectrometry	Following colloidal Coomassie Blue staining, gel lanes were cut into five pieces each and processed for liquid chromatography-mass spectrometry (LC-MS) analysis as described before (46).	Quantitative mass spectrometry analysis	https://www.ebi.ac.uk/pride/archive/projects/PXD061876	PXD061876	Properly shared on PRIDE	
Mass Spectrometry: Liquid Chromatography Mass Spectrometry	Following colloidal Coomassie Blue staining, gel lanes were cut into five pieces each and processed for liquid chromatography-mass spectrometry (LC-MS) analysis as described before (46).	Quantitative mass spectrometry analysis	https://www.ebi.ac.uk/pride/archive/projects/PXD061877	PXD061877	Properly shared on PRIDE	

No Action Required	*Optional*					
Quality control and/or representative media are recommended but not required to be uploaded to a repository						
**Datatype**	**Associated sentence from text**	**Article Subsection**	**URL**	**Accession/DOI/PID**	**DataSeer Notes**	**Author Response**
Tabular Data: Assay	The protein concentration was determined by the Bradford assay.	In vivo [35 S] methionine labe				
Image: Radiography	Equivalent protein amounts were separated on Tris-Tricine 10-18% g	In vivo [35 S] methionine labe				
Image: Electrophoresis	Finally, mitochondria were sedimented and the samples analyzed by	Downregulation of mtDNA-enc				
Image: Electrophoresis	Equivalent amounts of material were analyzed by SDS-PAGE and we	Native immunoprecipitation of				
Tabular Data	RNA quality control was assessed in a Fragment Analyzer.	RNA sequencing and analysis				
Image: Microscopy	Images were acquired using a spinning disk microscope (Molecular D	Immunofluorescence staining,				
Tabular Data	Cells were counted using the DAPI staining and the Stardist algorithr	Immunofluorescence staining,				
Tabular Data	Basal and maximal respiration were measured upon the addition of 3	Real-time respirometry				
Flow Cytometry	BD-Canto flow cytometer (Becton Dickinson) was used to record 10,(	Membrane potential measurer				
Tabular Data: Assay	Mitochondrial respiratory chain complex activities were measured us	Measurement of mitochondria				
Tabular Data	Autoradiographic and western blot signal intensities were quantified v	Quantification and statistical a				

Code availability

**No Action Required**	*Done - Shared/Cited*					
Research outputs are appropriately shared/cited						
**Name or Type of Code**	**Associated sentence from text**	**Article Subsection**	**URL**	**DOI**	**DataSeer Notes**	**Author Response**
R	The downstream analysis was performed in RStudio (R version 4.3.0) using packages from the Bioconductor repository (40, 41) and the Tidyverse suite.	RNA sequencing and analysis	https://doi.org/10.5281/zenodo.15260712		Properly shared on Zenodo	
Python	Data analyses for CYTB KD and ND2 KD as well as FLAG IP experiments were carried out using the autoprot package in Python).	RNA sequencing and analysis	https://doi.org/10.5281/zenodo.15241939		Properly shared on Zenodo	

Links or citations for software

No Action Required	*Optional*					
Software objects are recommended but not required to be uploaded to a repository						
**Name or Type of Software**	**Associated sentence from text**	**Article Subsection**	**URL**	**RRID**	**DataSeer Notes**	**Author Response**

nSolver	The acquired data were analyzed with nSolver software (nan	Mitochondrial RNA detection b				
RStudio	The downstream analysis was performed in RStudio (R versi	RNA sequencing and analysis				
Bioconductor	The downstream analysis was performed in RStudio (R versi	RNA sequencing and analysis				
Tidyverse	The downstream analysis was performed in RStudio (R versi	RNA sequencing and analysis				
DESeq2	Differential gene expression analysis was conducted using D	RNA sequencing and analysis				
limma	Batch correction was applied using limma (version 3.56.2) (4	RNA sequencing and analysis				
clusterProfiler	For Gene Ontology (GO) term enrichment analysis, the cluste	RNA sequencing and analysis				
org.Hs.eg.db	For Gene Ontology (GO) term enrichment analysis, the cluste	RNA sequencing and analysis				
UpSetR	UpSet plots were generated using UpSetR (version 1.4.0) co	RNA sequencing and analysis				
vsn	UpSet plots were generated using UpSetR (version 1.4.0) co	RNA sequencing and analysis				
ggplot2	Graphical representations were generated through the ggplot	RNA sequencing and analysis				
MaxQuant	The software MaxQuant/Andromeda (version 2.0.2.0 for COX	Quantitative mass spectromet				
UniProt	The software MaxQuant/Andromeda (version 2.0.2.0 for COX	Quantitative mass spectromet				
Perseus	The minimum peptide number required for LFQ was set to 1.	Quantitative mass spectromet				
impseq	The remaining missing values were imputed using sequential	Quantitative mass spectromet				
RankProd	Protein abundance ratios and p-values were calculated using	Quantitative mass spectromet				
Python	Data analyses for CYTB KD and ND2 KD as well as FLAG IP	Quantitative mass spectromet				
autoprot	Data analyses for CYTB KD and ND2 KD as well as FLAG IP	Quantitative mass spectromet				
ImageQuantTL	Autoradiographic and western blot signal intensities were qu	Quantification and statistical a				
ImageJ	Autoradiographic and western blot signal intensities were qu	Quantification and statistical a				
GraphPad Prism	Data were obtained from three or more biological replicates (	Quantification and statistical a				

To Be Completed by Authors

Design:

Experimental study design (statistics details)			
**For in vivo studies: State whether and how the following have been done**	**In vivo Study Information**	**Article Subsection**	**Author Response**
Sample size determination			
Randomisation			
Blinding			
Inclusion/exclusion criteria			

Sample definition and in-laboratory replication	Sample Information	Article Subsection	Author Response
State number of times the experiment was replicated in laboratory.			
Define whether data describe technical or biological replicates.			

Dual Use Research of Concern (DURC)	Authority and Reference Number	Article Subsection	Author Response
If study is subject to dual use research of concern regulations, state the authority granting approval and reference number for the regulatory approval.			

Analysis:

Attrition	Article Subsection	Author Response

Describe whether exclusion criteria were preestablished. Report if sample or data points were omitted from analysis. If yes report if this was due to attrition or intentional exclusion and provide justification.		

**Statistics**			
Describe statistical tests used and justify choice of tests.			
**Statistical Test**	**Justification**	**Article Subsection**	**Author Response**

Reporting

MDAR framework recommends adoption of discipline-specific guidelines, established and endorsed through community initiatives. Journals have their own policy about requiring specific guidelines and recommendations to complement MDAR.

**Adherence to community standards**	**Guideline/Checklist**	**Article Subsection**	**Author Response**
State if relevant guidelines (e.g., ICMJE, MIBBI, ARRIVE) have been followed, and whether a checklist (e.g., CONSORT, PRISMA, ARRIVE) is provided with the manuscript.			
