# Supplementary material for: Silencing mitochondrial gene expression in living cells
Source: Science. Author manuscript; Available in PMC 2025 Oct 18. (PMC7618265; doi:10.1126/science.adr3498)
Supplement: Supplementary Material [file EMS209270-supplement-Supplementary_Material.zip › science.adr3498_sm.pdf]

Science AAAS

Supplementary Materials for

Silencing mitochondrial gene expression in living cells

Luis Daniel Cruz-Zaragoza *et al*.

Corresponding authors: Luis Daniel Cruz-Zaragoza, luis.daniel.cruz.zaragoza@usherbrooke.ca; Peter Rehling, peter.rehling@medizin.uni-goettingen.de

Science **389**, eadr3498 (2025)
DOI: 10.1126/science.adr3498

The PDF file includes:

Figs. S1 to S7
References

Other Supplementary Material for this manuscript includes the following:

Tables S1 to S5
MDAR Reproducibility Checklist

Materials and Methods

Synthesis of Jac1- and peptide-morpholino oligonucleotide (MO) chimera

Jac1-MO chimeras were synthesized as previously described (*19*). Briefly, Jac1-DBCO (Dibenzocyclooctin) was mixed with an 8-fold molar excess of morpholino phosphorodiamidate oligonucleotides (MO) (Gene Tools) containing 5'-[azide] and 3'-[FITC] groups. The click reaction was carried out at 25°C for two hours with constant mixing. Peptide-MO chimeras were synthesized by a click chemistry reaction. pCox4$^{1-25}$ (*Saccharomyces cerevisiae* Cox4 presequence), containing a lysine-azide group at the C-terminus (MLSLRQSIRFFKPATRTLCSSRYLL{Lys(N_3)}-amide) (GenScript) was mixed in a molar ratio of 1:1 with morpholino phosphorodiamidate oligonucleotides (MO) (Gene Tools) containing 5'-[cyclooctyne] and 3'-[FITC] groups, final concentration of 100 μM. The click reaction was carried out at 20°C overnight, with periodic mixing (cycles of 30 sec mixing, 30 min rest). Chimeras used for transfection in cell culture were sterile-filtered with small 0.22 μm filters. The morpholinos used in this study were:

ND1$^{1-17}$, AGGTTGGCCATGGGTAT;
ND2$^{12-29}$, TAGATGACGGGTTGGGCC;
ND3$^{1-25}$, TTATTAAAATTAAGGCGAAGTTTAT;
ND4L$^{6-30}$, TAGTATAATATTTATGTAAATGAGG;
ND4$^{[-15]-10}$, GTTTTAGCATTGGAGTAGGTTTAGG;
ND5$^{3-25}$, TGGTTATAGTAGTGTGCATGGTT;
ND6$^{4-28}$, CACTCAACAGAAACAAAGCATACAT;
CYTB$^{6-24}$, GTTAGTTTTGCGTATTGGG;
COX1$^{1-19}$, GTCAACGGTCGGCGAACAT;
COX1$^{181-199}$, AGATTATTACAAATGCATG;
COX2$^{1-23}$, CCTACTTGCGCTGCATGTGCCAT;
COX3$^{2-19}$, CATGTGATTGGTGGGTCA;
ATP8$^{6-28}$, GCCATACGGTAGTATTTAGTTGG;
ATP6$^{[-10]-15}$, CAGATTTTCGTTCATTTTGGTTCTC;
mCOX1$^{1-24}$, GAATAATCAACGATTAATGAACAT;
xCOX1$^{1-22}$, ATAATCAACGAGTAATTGCCAT;
COX1$^{19-42}$, GTCTTTGTGGTTTGTAGAGAATAG;
COX1$^{40-64}$, ATAATAGGTATAGTGTTCCAATGTC;
COX1$^{65-82}$, CTCCAGCTCATGCGCCGA; and
COX1$^{85-104}$, AGGCTTAGAGCTGTGCCTAG.

The morpholino used to prepare the chimera Jac1-COX1$^{1-19}$ contained 5'-[azide] and 3'-[FITC] groups as previously (*19*).

Mammalian cell culture

HEK293-Flp-In™ T-Rex™ (HEK293T), HEK293T-derived, and HeLa cells were cultured in DMEM, supplemented with 10% (v/v) fetal bovine serum, 2 mM L-glutamine, 1 mM sodium pyruvate, and 50 μg/mL uridine. AML12 (alpha mouse liver 12) cells (CRL-2254™, ATCC) were cultured in DMEM:F12 media supplemented with 10% fetal bovine serum (FBS), 10 μg/ml insulin, 5.5 μg/ml transferrin, 5 ng/ml selenium, and 40 ng/ml dexamethasone. Human iPS cell-derived cardiomyocytes were cultured in RPMI 1640 (with GlutaMAX and HEPES) with the final concentration of 1× B-27 Supplement. All cells were incubated in a humidified incubator at 37°C

and 5% CO_2. HEK293T mL45FLAG cells (*19*) were treated with 0.6 mg/mL of tetracycline for 24 hours to induce the expression of mL45^{FLAG}.

***In vivo* [^{35}S]methionine labeling of mitochondrial translation products**

The medium of HEK293T, AML12, and cardiomyocytes cells was exchanged to DMEM methionine/cysteine-free supplemented with 2 mM L-glutamine, 1 mM sodium pyruvate, 50 µg/mL uridine, and 100 U/mL Pen/Strep, and incubated for 10 minutes. This step was repeated. Then DMEM methionine/cysteine-free supplemented with 10% (v/v) dialyzed fetal bovine serum, 2 mM L-glutamine, 1 mM sodium pyruvate, 50 µg/mL uridine, and 100 U/mL Pen/Strep was added to cells. The cytosolic translation was inhibited with 100 µg/mL emetine for 10 minutes. Next, 200 µCi/mL of [^{35}S] methionine were added, and the cells were incubated for one hour at 37°C with 5% CO_2. The cells were harvested, solubilized in Lysis buffer (50 mM Tris, 140 mM NaCl, 10 mM $MgCl_2$, 1% NP40, 1 mM PMSF, 1x Roche protease inhibitor cocktail), and the insoluble material removed by centrifugation at 5,000 xg for 5 min. The protein concentration was determined by the Bradford assay. Equivalent protein amounts were separated on Tris-Tricine 10-18% gradient gels, transferred to a PVDF membrane, and the radioactive signal detected by digital autoradiography.

mtDNA-encoded gene silencing in living cells

HEK293T, AML12, and HeLa cells were seeded in 12-well cell culture plates (80,000 cells/well), 6-well plates (16,0000 cells/well), and T25 cell culture flask (400,000 cells/flask). The cells were incubated for 24 hours at 37°C with 5% CO_2. For 72 hours silencing treatment, the media was exchanged to fresh media containing Endo-Porter (GeneTools) at 2 µM final concentration. Unless indicated otherwise, chimeras were added at 2 µM final concentration. For 24 and 48 hours treatments, the media was initially exchanged to fresh media. After 24 or 48 hours, the media was one more time exchanged, but to fresh media containing 2 µM Endo-Porter and 2 µM chimera to start the silencing, and incubated for 48 and 24 hours, respectively. The cells were then harvested or used for further analyses. Human iPS cell-derived cardiomyocytes (obtained from the Stem Cell unit at University Medical Center Goettingen) were seeded at a cell density of approximately one million cells per well in a 6-well plate. Media was exchanged to fresh media containing 4 µM Endo-Porter and 2.5 µM chimera, and incubated for 24 hours. The cells were then harvested or used for further analyses. The chimera and Endo-Porter concentrations have to be titrated for each cell type to guarantee efficient, robust, and reproducible mtDNA gene silencing.

siRNA-mediated protein knockdown

siRNAs targeting ZNF703, TMEM186, and *LINC00493* were purchased from Horizon Discovery (UK). HEK293T cells (1x10^6 cells) were transfected with Lipofectamine RNAiMAX (Invitrogen) following the manufacturer's instructions and transferred to a T25 cell culture flask. Cells were transfected for 72 hours and then used for further analyses. In experiments requiring mitochondria isolation, the transfection mix was scaled-up accordingly. siRNA was used at 33 nM final concentration in all experiments.

Transient expression of FLAG-tagged proteins

The open reading frame (ORF) of TMEM186 (NM_015421.4), ZNF703 (NM_025069.3), and SMIM26 (NM_001348957.2) inserted in pcDNA3.1 plasmid, in frame with a C-terminal FLAG tag, were purchased from GenScript (NL). For FLAG immunoisolation experiments, plasmids

were transfected using PEI (polyethylenimine) transfection reagent (Polysciences Europe). Briefly, HEK293T cells were seeded to a confluency of 50% two days prior to transfection in a 145 mm cell culture dish. 100 µL PEI (stock 10 mg/ml) were mixed with 400 µL OptiMEM, incubated at RT for 5minutes, and 4.5 mL of OptiMEM were added. For the transfection, 1 mL PEI/OptiMEM mix was transferred to a new tube containing 20 µg plasmid DNA in 1 mL OptiMEM, followed by mixing. After a 20 minutes incubation at RT, 10 mL of DMEM media supplemented with 10% (v/v) dialyzed fetal bovine serum, 2 mM L-glutamine, 1 mM sodium pyruvate, and 50 µg/mL uridine was added. The mixture was carefully transferred to the cell culture dish. After one hour incubation under standard growth condition, 10 mL of fresh media was added to the cells, and further incubated for 24 hours prior harvest.

Isolation of mitochondria

For *in organello* experiments (e.g. translation, import, gene expression silencing with chimera), mitochondria were isolated as previously described (*48*). Briefly, cells were washed with ice-cold isotonic buffer (10 mM MOPS pH 7.2, 225 mM sucrose, 75 mM mannitol, and 1 mM EGTA). After determining the cell pellet weight, it was resuspended in 5mL/(g cells) of cold hypotonic buffer (10 mM MOPS pH 7.2, 100 mM sucrose, and 1 mM EGTA) supplemented with 2 mM PMSF. The cell suspension was incubated on ice for 6 minutes and further homogenized in a glass-glass borosilicate homogenizer. Cold hypertonic buffer (1.25 M sucrose and 10 mM MOPS pH 7.2) was added to the cell homogenate 1.1 mL/(g cells), and the volume doubled with isotonic buffer supplemented with 2 mM PMSF and 2 mg/mL BSA. Unbroken cells and cell debris were removed by centrifugation at 1,000 x g for 10 minutes at 4°C, the supernatant recovered, and the same step repeated. Mitochondria were pelleted by centrifuging the supernatant at 11,000 xg for 10 minutes at 4°C. The pellet was resuspended in isotonic buffer without BSA and sedimented (for washing). Finally, the pellet was resuspended in isotonic buffer, and the protein concentration of isolated mitochondria was determined using the Bradford assay.

HEK293T mitochondria required for steady-state analyses (SDS- and BN-PAGE), mass spectrometry, and immunoprecipitation (for western blot and nanoString analyses) were isolated using a teflon-glass homogenizer. Briefly, the cell pellet was resuspended in THE buffer (10 mM HEPES/KOH, 300 m Trehalose, 10 mM KCl, 1 mM EGTA, pH 7.4) supplemented with 1 % BSA and 1mM PMSF, and incubated for 10min on ice. The suspension was homogenized by strokes at 800 rpm. Cell debris and unbroken cells were removed by centrifugation, first, at 400 xg, 10 minutes, 4°C, and then at 800 xg, 10 minutes, 4°C. Mitochondria were sedimented at 11,000 xg, 10 minutes, 4°C. After discarding the supernatant, the mitochondrial pellet was washed by resuspension in THE buffer, and the protein concentration of isolated mitochondria was determined using the Bradford assay.

For mitochondrial immunoisolation, the Mitochondria Isolation Kit, Human (Miltenyi Biotech, Catalog No: 130-094-532) was used. The experiment was conducted according to the manufacturer's protocol using µ Columns, except additional washes and the isolation of mitochondria in an isotonic buffer (75 mM mannitol, 225 mM sucrose, 10 mM MOPS, pH 7.2, and 1 mM EGTA). Sub-mitochondrial protein localization studies were performed as previously described (*19*).

Downregulation of mtDNA-encoded genes by chimera in purified mitochondria

The downregulation of mtDNA-encoded gene expression was performed as previously described (*19*). In brief, freshly isolated mitochondria (human and mouse) were resuspended at 1 mg/mL

protein concentration in import buffer (20 mM HEPES, 100 mM mannitol, 80 mM KCl, 5 mM $MgCl_2$, 10 mM sodium succinate, 10 mM malate, 5 mM ATP, 6 mM creatine phosphate, 0.625 mg/mL creatine kinase, 1 mg/mL BSA, 5 mM NADH, pH 7.4). The import reaction was started by adding 2 μL per 100 μL import mix of the chimera at the appropriate final concentration (serial dilutions for the titration experiments, or 0.25 μM). To the positive translation control (no silencing), 20 mM HEPES, 100 mM mannitol, pH 7.4 buffer or non-targeting chimera were added. The mixes were incubated for 20 minutes at 37°C under mild shaking conditions Mitochondria were sedimented at 11,000 xg for 10 minutes at 4°C. Next, the mitochondria were resuspended at 1 mg/mL protein concentration in freshly prepared translation buffer (25 mM HEPES, 100 mM mannitol, 80 mM KCl, 5 mM $MgCl_2$, 10 mM sodium succinate, 1 mM potassium phosphate, 5 mM ATP, 6 mM creatine phosphate, 0.625 mg/mL creatine kinase, 1 mg/mL BSA, 0.02 mM GTP, 0.15 mM amino acid mix (minus methionine), 100 µg/mL emetine, pH 7.4). The negative control for the translation reaction was obtained by adding 0.3 mg/mL chloramphenicol. Samples were incubated for 5 minutes at 37°C, 100 µCi/mL of [^{35}S] methionine were added, and the translation was performed for one hour at 37°C. When required, the translation was stopped by adding 0.3 mg/mL chloramphenicol. Finally, mitochondria were sedimented and the samples analyzed by SDS-PAGE in Tris-Tricine 10-18% gradient gel followed by digital autoradiography.

Native immunoprecipitation of FLAG-tagged proteins
Protein complex isolation by FLAG immunoisolation was performed as previously described (*19*). Briefly, mitochondria or cells were solubilized at 1 mg/mL protein concentration in lysis buffer (50 mM Tris/HCl pH 7.4, 150 mM NaCl, 10% glycerol, 10 mM $MgCl_2$, 1% Digitonin, 1 mM PMSF, 1x complete protease inhibitor cocktail, 0.08 U/µL RiboLock RNase inhibitor) for 30 minutes on ice with periodic mixing. The insoluble material was removed by centrifugation at 10,000 xg for 10 minutes at 4°C. The lysate was then mixed with anti-FLAG M2 Affinity Gel (Sigma-Aldrich) and incubated for one hour at 4°C. Next, the unbound fraction was removed and the resin washed with washing buffer (50 mM Tris/HCl pH 7.4, 150 mM NaCl, 10% glycerol, 10 mM $MgCl_2$, 0,1% Digitonin, 1 mM PMSF, 1x complete protease inhibitor cocktail, 0.08 U/µL RiboLock RNase inhibitor). The native protein complexes were eluted with 0.4 mg/mL FLAG peptide in washing buffer for 30 minutes at 4°C. HEK293T-derived mitochondria were used as negative controls. Equivalent amounts of material were analyzed by SDS-PAGE and western blotting. For RNA isolation and nanoString analysis, the input and eluate samples were mixed with 1 mL Trizol reagent (Thermo Scientific Fisher) and processed as described below.

Mitochondrial RNA detection by nanoString technology
Immunoisolation input and eluate fractions were mixed with Trizol reagent (Thermo Scientific Fisher) and the RNA purified using RNA Clean & Concentrator kit (Zymo Research) following the manufacturer’s instructions. For the analysis of siRNA-treated cells, mitochondria were isolated and solubilized in immunoprecipitation’s lysis buffer with 1% digitonin as described above. The cleared lysate was then processed for RNA isolation. Equivalent amounts of RNA were mixed with a TagSet-24 and detection primers (IDT) previously used to detect mitochondrial transcripts (*19*, *38*). Next, the samples were processed and analyzed in a nCounter® MAX analysis system (nanoString) following the manufacturer’s instructions. The acquired data were analyzed with nSolver software (nanoString).

RNA sequencing and analysis

HEK293T cells were treated for 48 hours with chimeras targeting different mtDNA-encoded mRNAs. Control samples were transfected with pCox4 in the click reaction buffer. After discarding the media, cells were resuspended in Trizol reagent. The RNA was purified using RNA Clean & Concentrator kit (Zymo Research) following the manufacturer's instructions. RNA quality control was assessed in a Fragment Analyzer. The NovaSeq X Plus sequencing platform (Illumina, USA) was used to perform 50 bp paired-end sequencing on the samples with 9 G raw data per sample. For each treatment, four biological replicates were analyzed, except for COX2 and ND2 where three biological replicates prepared and measured.

The downstream analysis was performed in RStudio (R version 4.3.0) using packages from the Bioconductor repository (*39*, *40*) and the Tidyverse suite.

Differential gene expression analysis was conducted using DESeq2 (version 1.40.2) (*41*). Batch correction was applied using limma (version 3.56.2) (*42*) to account for variability between replicates performed on different days with different reagent batches (batch1: replicate 1 and 2; batch 2: replicate 3 and 4) to improve clustering and reliability of differential expression results.

For Gene Ontology (GO) term enrichment analysis, the clusterProfiler (version 4.8.3) (*43*) package was used, with annotations from org.Hs.eg.db (version 3.17.0), focusing on Biological Processes (BP) and Molecular Functions (MF). UpSet plots were generated using UpSetR (version 1.4.0) (*44*) to visualize intersections of significantly changed genes across conditions. Graphical representations were generated through the ggplot2 package (version 3.5.1).

Protein electrophoresis and immunodetection

Tris-Tricine 10-18% gradient gel electrophoresis was used to analyze the samples obtained in downregulation of mitochondrial translation experiments and samples from immunoisolation experiments. Native complexes solubilized in solubilization buffer (20 mM Tris/HCl pH 7.4, 150 mM NaCl, 10% glycerol, 1 mM EDTA, 1 mM PMSF, 1x complete protease inhibitor cocktail) containing 1 % digitonin or 1 % DDM were resolved by 4-13% Blue Native (BN)-PAGE. For two dimensional analyses, the native complexes solubilized in digitonin were resolved in 4-13% BN-PAGE gels. Each lane was cut, assembled in a Tris-Tricine 10-18% gradient gel, and the proteins resolve by SDS-PAGE. After the electrophoresis, the proteins were transferred to PVDF membranes, exposed for autoradiography, and processed for western blotting.

Quantitative mass spectrometry analysis

Mitochondria-enriched fractions prepared from cells treated with pCox4-COX1$^{1\text{-}19}$ chimera (*COX1*$^{1\text{-}19}$ or COX1^{KD}) and pCox4 (Control), silencing for 8, 16, 24, 48, and 72 hours (4 independent replicates each); as well as pCox4-ND1$^{2\text{-}29}$ (*ND2*$^{12\text{-}29}$ or ND2^{KD}), pCox4-CYTB$^{6\text{-}24}$ (*CYTB*$^{6\text{-}24}$ or CYTB^{KD}), and pCox4 (Control) cells (n=4 each) were lysed in urea buffer (8 M urea in 50 mM ammonium bicarbonate). Cysteine residues were reduced and free thiol groups alkylated by incubation with 5 mM Tris(2-carboxy-ethyl)phosphine and 50 mM iodoacetamide for 1 h at 37°C. The reaction was quenched by addition of dithiothreitol (25 mM final concentration). Samples were diluted with 50 mM ammonium bicarbonate to reach a urea concentration of 1 M, followed by tryptic protein digestion (overnight, 37°C) at a trypsin-to-protein ratio of 1:50. Proteins of ZNF703^{FLAG}, TMEM186^{FLAG}, SMIM26^{FLAG} and control (HEK293T WT) eluates of FLAG-immunoisolation experiments (n=3 each) were separated by SDS-PAGE. Following colloidal Coomassie Blue staining, gel lanes were cut into five pieces each and processed for liquid chromatography-mass spectrometry (LC-MS) analysis as described before (*50*). Prior to LC-MS

analysis, peptides of slices number 1, 2 and 4 were combined. Peptide mixtures of all experiments were desalted using StageTips (*27*) and analyzed on a Q Exactive Plus instrument (Thermo Fisher Scientific, Germany). The software MaxQuant/Andromeda (version 2.0.2.0 for $COX1^{KD}$, 2.6.5.0 for FLAG IP and 2.6.6.0 for $CYTB^{KD}$ and $ND2^{KD}$ samples; (*50*)) and the UniProt human ProteomSet (including isoforms) were used for protein identification applying MaxQuant default settings. For the analysis of $COX1^{KD}$, $CYTB^{KD}$ and $ND2^{KD}$ samples, the option 'match between runs' was enabled. Relative protein quantification was performed using the 'LFQ' algorithm (i.e., label free quantification; $COX1^{KD}$, $CYTB^{KD}$ and $ND2^{KD}$ samples) or 'iBAQ' values (FLAG IPs) (*50*). The minimum peptide number required for LFQ was set to 1. Missing LFQ intensities of $COX1^{KD}$ experiments were replaced from a normal distribution using Perseus (version 1.6.2.2; (*49*)), applying a width of 0.3 and a down shift of 2.5. For each time-point, the mean of $\log_2$ LFQ intensity ratios ($COX1^{KD}$ v*ersus* control) was determined, and p-values were calculated using a one-sample two-sided Student's t-test. Results for all time points of $COX1^{KD}$ experiments are provided in supplemental Table S4. For the analysis of $CYTB^{KD}$ and $ND2^{KD}$ experiments, data were filtered to protein groups with LFQ intensities in at least three replicates. The remaining missing values were imputed using sequential imputation (R package 'impseq'; (*50*) and the resulting distributions were normalized using cyclic LOESS (R package 'limma'; (*45*). Protein abundance ratios and p-values were calculated using rank sum analysis (R package 'RankProd'; (*52*)). Results of $CYTB^{KD}$ and $ND2^{KD}$ experiments are provided in supplemental Table S3. For the analysis of FLAG IP experiments, MaxQuant iBAQ values of the different gel slices of a replicate were summed and the resulting intensities were normalized using variance-stabilization across the replicates of a bait protein or the control experiment (R package vsn; (*53*)). Missing values were imputed with the median within the corresponding protein group for the control experiment and by sampling from a normal distribution with 0.3 width and a single standard deviation downshift for the immunoisolated samples. Statistics were computed using rank sum analysis as described above. For results of FLAG IP experiments, see supplemental Table S5. Data analyses for $CYTB^{KD}$ and $ND2^{KD}$ as well as FLAG IP experiments were carried out using the autoprot package in Python).

Immunofluorescence staining, imaging, and analysis
HeLa cells were seeded on a 24-well glass bottom slide (5,000-10,000 cells per well), and incubated for 24 hours. The medium was exchanged after 24 hours with fresh medium containing EndoPorter (no EndoPorter in the non-transfected control) and the respective compound undy study (e.g., pCox4-$COX1^{1\text{-}19}$ chimera, $COX1^{1\text{-}19}$ morpholino, etc.) at a final concentration of 2 µM. The compounds were applied for 24 or 72 hours. By initiating the treatment at different starting time points, all transfections were stopped simultaneously by rinsing the wells with PBS. The cells were then chemically fixed with 8% formaldehyde in PBS at 37°C for 5 min. Afterwards, permeabilization was performed using 0.5% Triton-X-100 in PBS for 10 min. Following a washing step with PBS, the primary antibody solution was applied for one hour (1:200 anti-TOM20 antibody from rabbit (Abcam) in PBS). After three washing steps with PBS, the secondary antibody solution was applied for one hour (1:300 anti-rabbit antibody from goat (AffiniPure) coupled with StarRed (abberior) and 1:10,000 DAPI–dihydrochlorid (Sigma-Aldrich in PBS)). Subsequently three washing steps with PBS were performed. Images were acquired using a spinning disk microscope (Molecular Devices) with a 40x Plan Apo Lambda objective (Molecular Devices) and a 51 µm pinnhole disk (Molecular Devices). A 18x18 grid of images (each image has a xy-dimension of 350,82 µm x 350,82 µm) was recorded per well. A z-stack of 10 image

planes (with 200 µm z-steps) was recorded and a maximum projection was generated. In order to compare recordings with similar confluency, only images with 5 – 60 cells were analyzed.

For the analysis, images were first denoised using N2V2 (*54*) trained on a random subset of images to remove technical noise. Cells were counted using the DAPI staining and the Stardist algorithm (*55*) with a threshold of 0.5 and filtering to objects of typical HeLa nuclei size (110-553 μm^2). The segmented nuclei were dilated by 3 pixels and the obtained segmented nucleus area was deleted from all channels for subsequent analysis. Mitochondrial structures were detected using a threshold of 120 on the TOM20 images after z-normalisation and conversion to 8 bit between -1 and 6σ. The mitochondrial network was analyzed after skeletonization by scikit-image (*56*). Mitochondrial length was defined as the mitochondrial segment between two branching points of the mitochondrial network. The boxplot was created by weighing the average mitochondrial fragment length with the number of cells detected in the image.

Real-time respirometry

Seahorse XF96e Extracellular Flux Analyzer was used to measure the oxygen consumption rate (OCR) in HEK293T cells. Instructions provided by the manufacturer in the Seahorse XF Cell Mito Stress Test Kit User Guide were applied. Following a three-day knockdown transfection, the cells were counted and resuspended in XF DMEM buffer + 1 mM pyruvate, 2 mM glutamine, and 10 mM glucose. 50,000 cells were plated on a Seahorse XF cell culture plate. Once plated, the cells were incubated in a non-CO_2 incubator for one hour before proceeding for measurement. Basal and maximal respiration were measured upon the addition of 3 mM Oligomycin, 1.5 mM CCCP, and 0.5 mM Antimycin/Rotenone. The measurements were normalized to the cell number.

The extracellular acidification rate (ECAR) of treated HEK293T cells was measured as instructed in the Seahorse XF Glycolysis Stress Test Kit User Guide (Agilent Technologies). 50,000 cells were plated on a Seahorse XF cell culture plate for this assay in XF DMEM Buffer (supplemented with 1 mM pyruvate and 2 mM glutamine). Basal acidification was measured, followed by consequent measurements upon the addition of 10 mM Glucose 3 µM Oligomycin, and 50 mM 2-deoxy-D-glucose. The measurements were normalized to the cell number. Cell numbers were determined using CyQUANT™ following the instructions provided by the manufacturer (Thermo Fisher Scientific).

Membrane potential measurement

TMRM (Invitrogen) was used to measure mitochondrial membrane potential. The HEK293T cells were stained with TMRM at a concentration of 10 µM, following the instructions provided by the manufacturer. BD-Canto flow cytometer (Becton Dickinson) was used to record 10,000 gated events per sample. The results were analyzed using the FACS-Diva software.

Measurement of mitochondrial complex I, III, IV and V activities in HEK293T cells

HEK293T cells were transfected with chimeras for 48 hours. Mitochondrial respiratory chain complex activities were measured using enzyme activity microplate assay kits from Abcam: Complex I Enzyme Activity Microplate Assay Kit (ab109721), Mitochondrial Complex III Activity Assay Kit (ab287844), Complex IV Rodent Enzyme Activity Microplate Assay Kit (ab109911), and ATP Synthase (Complex V) Enzyme Activity Microplate Assay Kit (ab109714). The assays were performed according to the manufacturer's instructions, using HEK293T cell lysates for complexes I, IV, and V, and isolated mitochondria from HEK293T cells for complex III. All samples were kept on ice throughout processing. For complex I measurements, 100 µg

protein of cell lysate were loaded per well in a 96-well plate; for complex III, 5 µg isolated mitochondria were used per reaction; in case of complex IV, 20 µg protein of cell lysate were used; for complex V, 50 µg protein of cell lysate were used per reaction. All absorbance measurements were conducted using a Synergy H1 microplate reader (BioTek). Enzymatic activities were calculated by determining the rate of absorbance change over time.

Quantification and statistical analysis

Autoradiographic and western blot signal intensities were quantified with ImageQuantTL v8.1 (GE Healthcare) and ImageJ v1.47 (NIH). Data were obtained from three or more biological replicates (n), and were processed with GraphPad Prism 8 software for statistical purposes. Mean, SEM, and statistical significance are listed in figure legends.

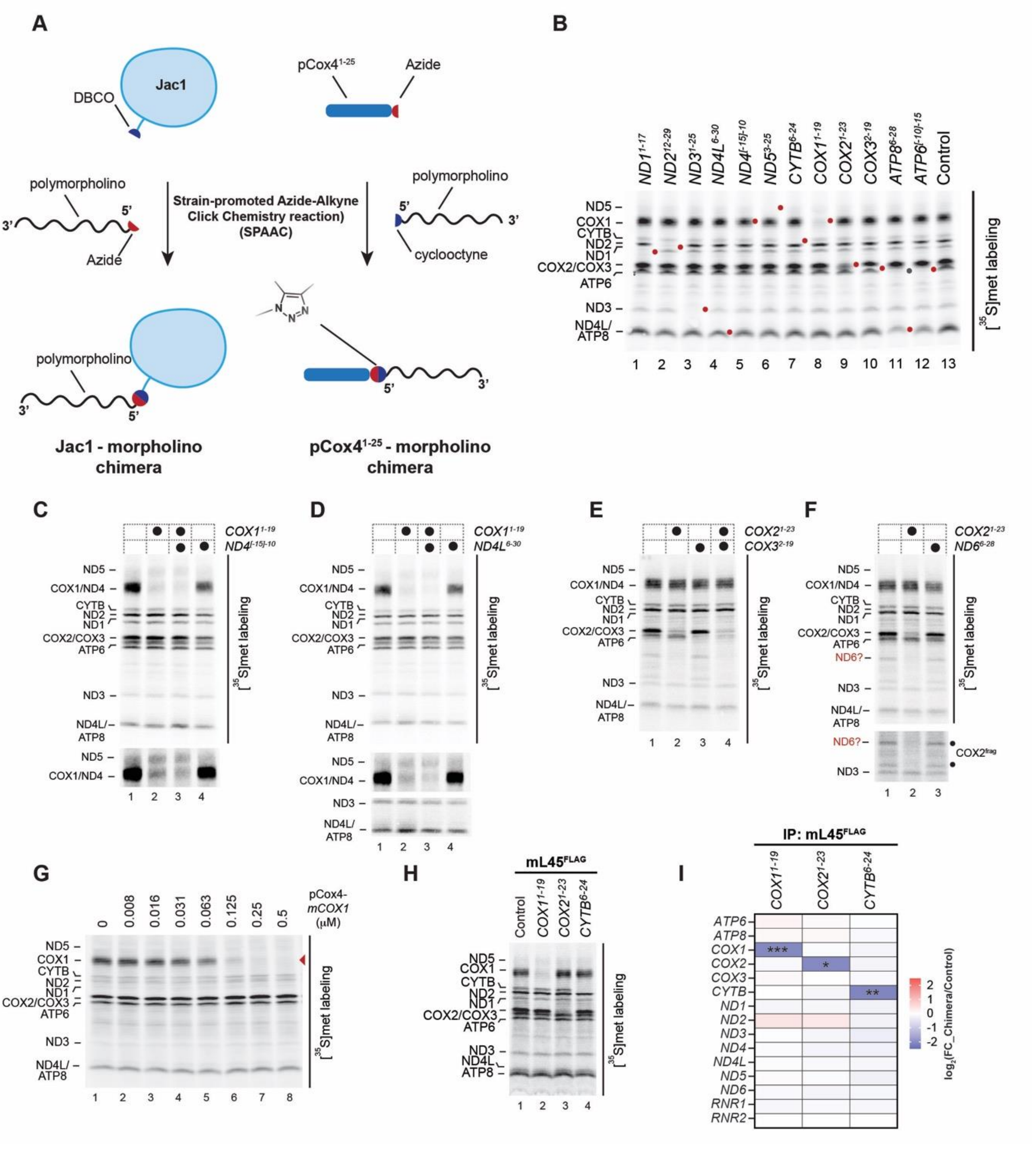

Fig. S1.

Gene silencing by peptide-morpholino chimeras in isolated mitochondria and living cells. (**A**) Jac1-MO and pCox4-MO chimera synthesis diagram. (**B-F**) *In organello* translation after downregulation of all mtDNA-encoded proteins (except ND6). Peptide-morpholino chimeras were synthesized and imported into isolated mitochondria, mitochondria reisolated, and subjected to [^{35}S]methionine labeling of translation products. Position of targeted proteins indicated by red dot.; position of ATP6 upon ATP8 silencing indicated by a gray dot (**B**). To visualize ND4 silencing with the chimeras targeting ND4 (**C**) and ND4L (**D**) mRNAs, we removed the signal of newly-synthesized COX1 by double knowdown. Since in our system the newly-synthesized COX2, COX3, and ATP6 migrated close together, the COX3 signal is partially hidden (**B**). Therefore, we

performed double knowdown experiments of COX2 and COX3 translation where the COX3 silencing is easily observed (**E**). The silencing of ND6 expression remained challenging. Strikingly, the newly-synthesized protein commonly referred to as ND6, represents a COX2 translation intermediate (indicated in the cropped section with a dot) (**F**). (**G**) Titration of pCox4-m$COX1^{1\text{-}24}$ chimera effect on protein translation in mitochondria isolated from AML12 cells (mouse hepatocytes). After chimera import, the newly synthesized mitochondrial proteins were labeled with [^{35}S]methionine. Samples were analyzed by SDS-PAGE followed by digital autoradiography. COX1 signal is indicated by an arrowhead. (**H-I**) Specificity of the silencing effect of chimeras imported into mitochondria is mediated by the impairment for the mitochondrial ribosome to bind to the targeted mRNAs. Mitochondria isolated from mL45^{FLAG}-expressing cells were treated with chimeras targeting the 5' region of the COX1, COX2, and CYTB mRNAs. [^{35}S]methionine labeling of translation products showed the silencing (**H**). Alternatively, upon import and translation, FLAG-IP was performed, RNA purified from the eluate fractions, and analyzed by nanoString. mRNA binding to the ribosome was significantly reduced. As shown in the heatmap (I), the amount of the cognate mRNAs, but not the other mtDNA-encoded mRNAs (mt-mRNA) and rRNAs (mt-rRNA, *RNR1* and *RNR2*), changed upon treatment compared to the control (n=3, mean). FC, fold change. Statistical significance was determined by multiple t test using the Holm-Sidak method, with alpha=0.05 (*, padj<0.05; **, padj<0.01; ***, padj<0.001). Non-significant differences are not indicated.

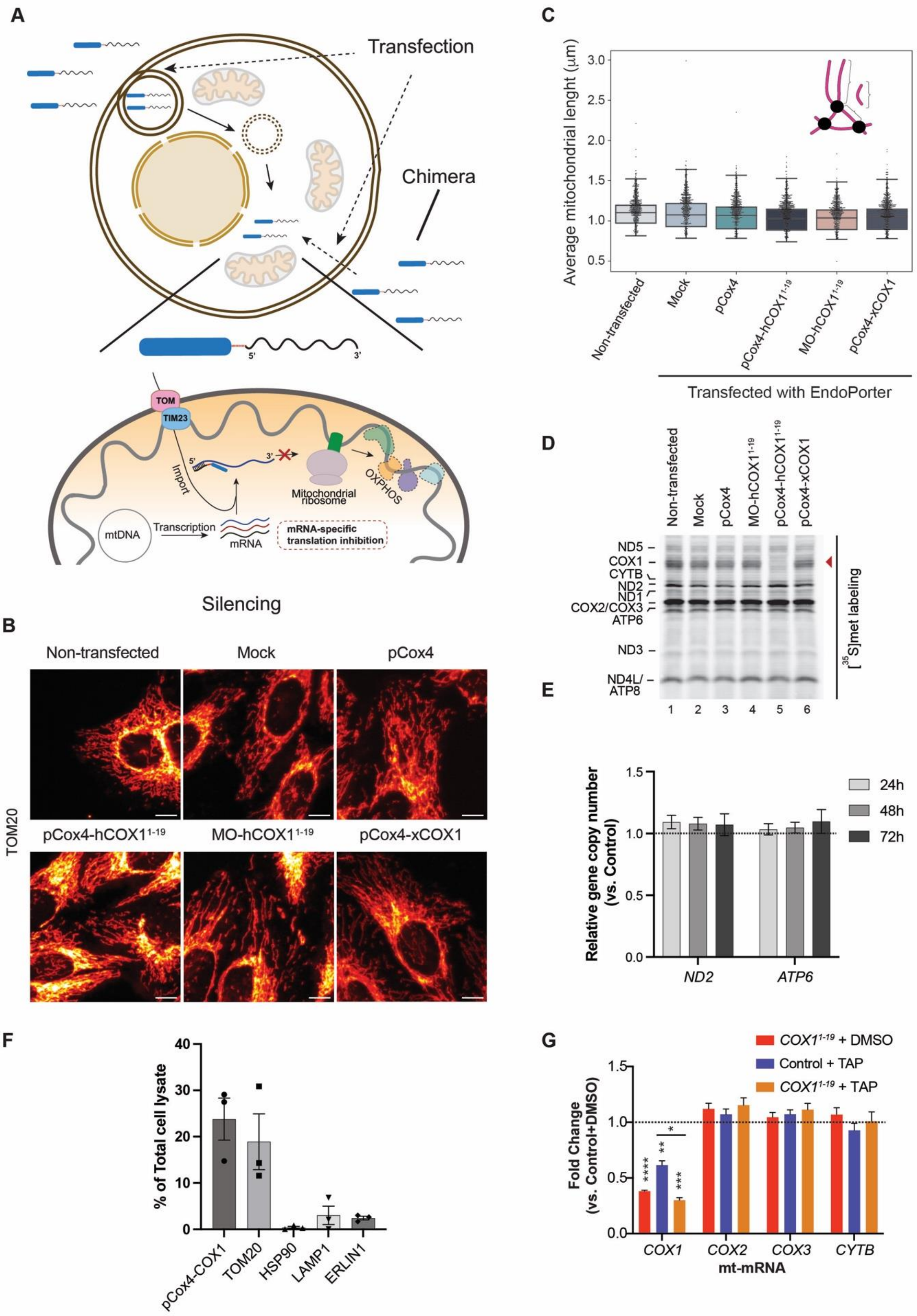

A
Transfection
Chimera
TOM
TIM23
5'
3'
Import
Mitochondrial ribosome
OXPHOS
mtDNA
Transcription
mRNA
mRNA-specific translation inhibition
Silencing
B
Non-transfected
Mock
pCox4
pCox4-hCOX1 1-19
MO-hCOX1 1-19
pCox4-xCOX1
TOM20
C
Average mitochondrial lenght (μm)
Non-transfected
Mock
pCox4
pCox4-hCOX1 1-19
MO-hCOX1 1-19
pCox4-xCOX1
Transfected with EndoPorter
D
Non-transfected
Mock
pCox4
MO-hCOX1 1-19
pCox4-hCOX1 1-19
pCox4-xCOX1
ND5
COX1
CYTB
ND2
ND1
COX2/COX3
ATP6
ND3
ND4L/ ATP8
[35S]met labeling
1 2 3 4 5 6
E
Relative gene copy number (vs. Control)
24h
48h
72h
ND2
ATP6
F
% of Total cell lysate
pCox4-COX1
TOM20
HSP90
LAMP1
ERLIN1
G
COX1 1-19 + DMSO
Control + TAP
COX1 1-19 + TAP
Fold Change (vs. Control+DMSO)
COX1
COX2
COX3
CYTB
mt-mRNA

Fig. S2.

Transfection of presequence-morpholino chimera in living cells. (**A**) Schematic representation of chimera uptake by living cells. After release from lysosomes, the cytosolic chimera is imported into the mitochondrial matrix, a process that is facilitated by the Cox4 presequence (pCox4$^{1-25}$). Once inside mitochondria, the morpholino binds to the target RNA to block translation. (**B**) Mitochondrial network analysis after cell transfection with chimeras and controls in HeLa cells. The treatment did not significantly impact mitochondrial morphology. HeLa cells were transfected for 24 hours with chimera targeting human *COX1* mRNA (pCox4-hCOX1$^{1-19}$), the unmodified presequence peptide (pCox4), free morpholino (MO-hCOX1$^{1-19}$), a chimera targeting *Xenopus laevis COX1* mRNA (pCox4-xCOX1), and buffer (Mock). Non-transfected cells were also used as reference. Mitochondria were labeled by anti-TOM20 antibodies. Scale bars, 5 μm. (**C**) Analysis of the average mitochondrial length obtained from (B). (**D**) [^{35}S]methionine labeling of mitochondrial translation products in cells after treatment as in (B). Newly-synthesized COX1 is indicated with an arrowhead. (**E**) COX1 silencing in cells did not significantly alter mtDNA copy number. (**F**) Presequence-morpholino chimeras are enriched in mitochondria. Upon transfection of pCox4-COX1$^{1-19}$ for 72 hours in HEK293T cells, mitochondria were immunoisolated with anti-TOM20 magnetic beads. Immunoisolation efficiency was assessed by Western blotting and quantified. Chimera enrichment is similar to that of TOM20. HSP90 (cytosol), LAMP1 (lysosome), and ERLIN (ER) were used as markers for other cellular compartments. (n=3; mean ± SEM). (**G**) *COX1* mRNA reduction is linked to mitochondrial translation. Cells were treated with thiamphenicol (TAP) to inhibit mitochondrial translation or with DMSO (control) for two hours. Then pCox4-COX1$^{1-19}$ chimera were transfected for 16 hours in the presence of TAP. Total RNA was purified from isolated mitochondria and analyzed by nanoString to determine the abundance of mRNAs. Only *COX1* mRNA was reduced after TAP treatment, and further decreased with chimera treatment (n=3; mean ± SEM). Statistical significance was determined by multiple t test using the Holm-Sidak method, with alpha=0.05 (*, padj<0.05; **, padj<0.01; ***, padj<0.001; ****, padj<0.0001).

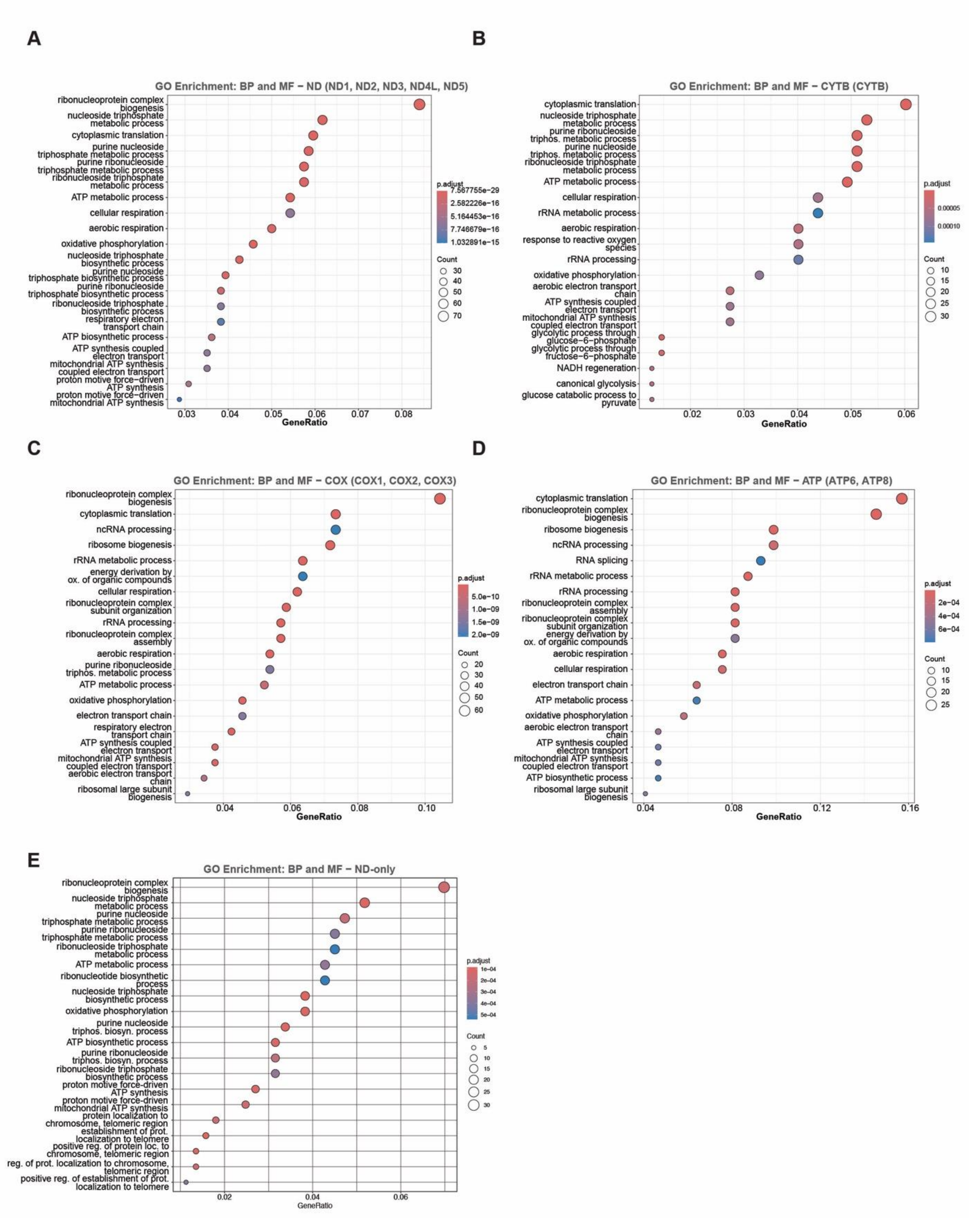

Fig. S3.

Changes in mRNA abundance upon silencing of mitochondrial mRNAs. (**A**) (**A-D**) Gene functional enrichment analysis by gene ontology (GO) annotation of mRNAs. mRNAs with significantly altered abundance (padj<0.05) from each treatment were grouped based on the

OXPHOS complex to which the silenced protein belongs: ND (complex I: ND1, ND2, ND3, ND4L, and ND5) (**A**), CYTB (complex III: CYTB) (**B**), COX (complex IV: COX1, COX2, and COX3) (**C**), and ATP (complex V: ATP6 and ATP8) (**D**). GO enrichment analysis was performed focusing on biological process (BP) and molecular function (MF). (**E**) Gene functional enrichment analysis by gene ontology annotation of mRNAs that significantly varied only upon silencing of complex I subunits (ND: $ND1 \cup ND2 \cup ND3 \cup ND4L \cup ND5$).

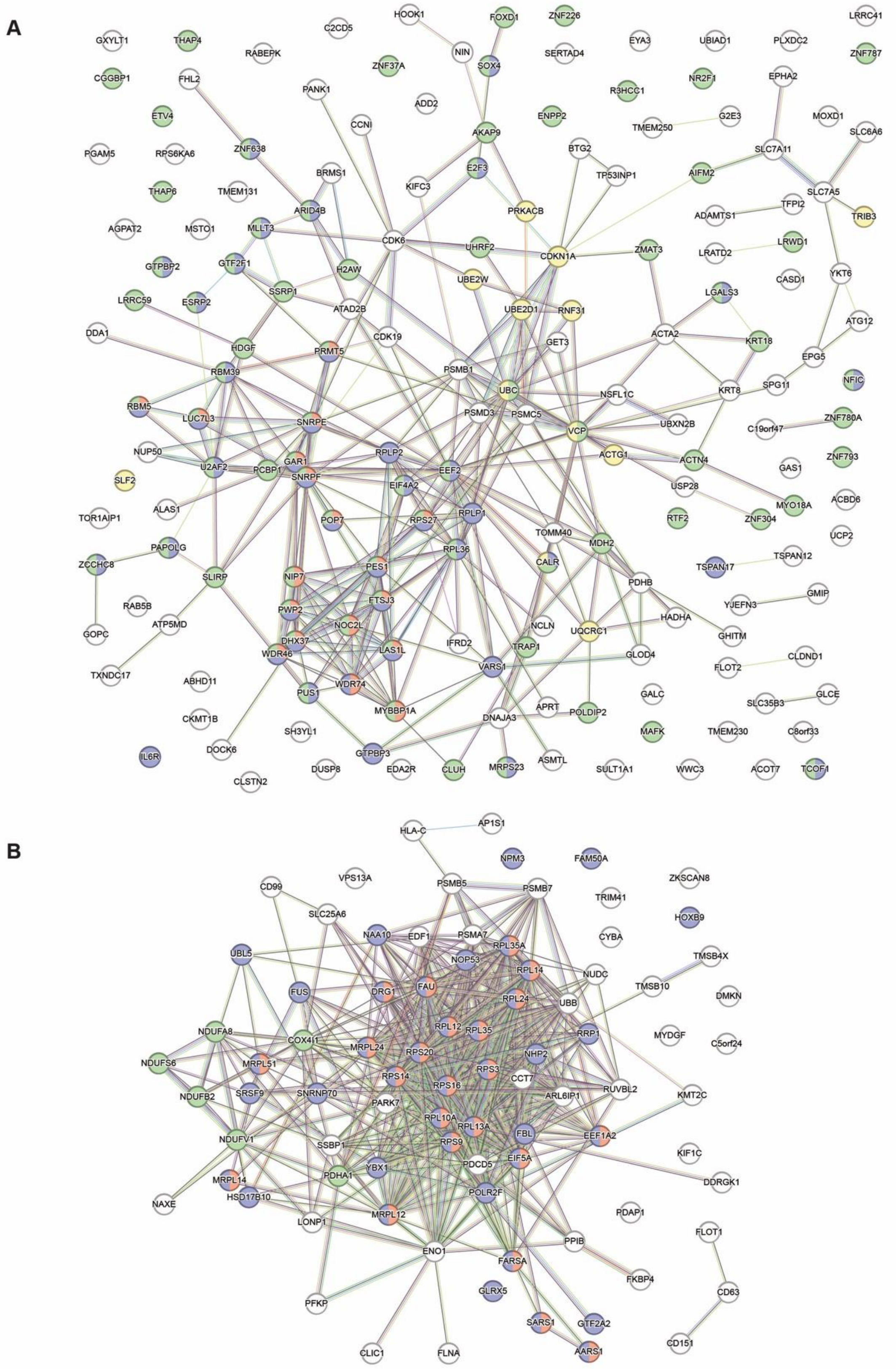
A
HOOK1
FOXD1
ZNF226
LRRC41
GXYLT1
THAP4
C2CD5
NIN
EYA3
UBIAD1
PLXDC2
RABEPK
SERTAD4
ZNF787
CGGBP1
FHL2
ZNF37A
SOX4
R3HCC1
NR2F1
EPHA2
PANK1
ADD2
ETV4
ENPP2
TMEM250
G2E3
MOXD1
CCNI
AKAP9
SLC6A6
PGAM5
RPS6KA6
ZNF638
BTG2
SLC7A11
BRMS1
KIFC3
E2F3
TP53INP1
AIFM2
THAP6
TMEM131
PRKACB
SLC7A5
TFPI2
ADAMTS1
TRIB3
AGPAT2
MSTO1
MLLT3
ARID4B
CDK6
UHRF2
CDKN1A
ZMAT3
LRATD2
LRWD1
GTPBP2
GTF2F1
H2AW
UBE2W
CASD1
YKT6
SSRP1
LGALS3
LRRC59
ESRP2
ATAD2B
UBE2D1
RNF31
ATG12
DDA1
CDK19
GET3
ACTA2
KRT18
HDGF
PRMT5
EPG5
PSMB1
RBM39
UBC
NSFL1C
KRT8
SPG11
NFIC
RBM5
LUC7L3
SNRPE
PSMD3
PSMC5
UBXN2B
C19orf47
ZNF780A
NUP50
VCP
ACTG1
ZNF793
RPLP2
U2AF2
PCBP1
GAR1
ACTN4
SLF2
SNRPF
EEF2
EIF4A2
USP28
GAS1
ACBD6
TOR1AIP1
ALAS1
POP7
RPS27
RPLP1
MYO18A
RTF2
ZNF304
UCP2
TOMM40
PAPOLG
RPL36
MDH2
ZCCHC8
CALR
TSPAN17
TSPAN12
SLIRP
NIP7
PES1
PDHB
RAB5B
FTSJ3
PWP2
YJEFN3
GMIP
ATP5MD
NOC2L
NCLN
HADHA
GHITM
GOPC
DHX37
UQCRC1
WDR46
LAS1L
IFRD2
TRAP1
GLOD4
CLDND1
TXNDC17
VARS1
FLOT2
ABHD11
WDR74
PUS1
APRT
GALC
SLC35B3
GLCE
CKMT1B
MYBBP1A
DNAJA3
POLDIP2
SH3YL1
MAFK
TMEM230
C8orf33
DOCK6
IL6R
GTPBP3
DUSP8
EDA2R
CLUH
MRPS23
ASMTL
SULT1A1
WWC3
ACOT7
TCOF1
CLSTN2
B
HLA-C
AP1S1
NPM3
FAM50A
CD99
VPS13A
PSMB5
PSMB7
ZKSCAN8
TRIM41
SLC25A6
HOXB9
CYBA
NAA10
EDF1
PSMA7
RPL35A
UBL5
TMSB4X
NOP53
RPL14
NUDC
FUS
DRG1
FAU
TMSB10
DMKN
RPL24
UBB
RPL12
RPL35
RRP1
MYDGF
NDUFA8
COX4I1
C5orf24
MRPL24
RPS20
NDUFS6
MRPL51
RPS3
NHP2
RPS14
RPS16
CCT7
SRSF9
SNRNP70
RUVBL2
KMT2C
NDUFB2
PARK7
ARL6IP1
RPL10A
RPL13A
FBL
EEF1A2
NDUFV1
SSBP1
RPS9
KIF1C
EIF5A
PDCD5
YBX1
PDHA1
DDRGK1
MRPL14
POLR2F
HSD17B10
NAXE
PDAP1
MRPL12
LONP1
FLOT1
PPIB
ENO1
FARSA
FKBP4
CD63
PFKP
GLRX5
SARS1
GTF2A2
CD151
CLIC1
FLNA
AARS1

Fig. S4.

Interaction network of proteins encoded by mRNAs in COX and ND,_CYTB,_COX,_ATP exclusive intersections. The group of mRNAs forming the exclusive intersections COX-only (**A**) and ND_CYTB_COX_ATP (**B**) were analyzed using the STRING algorithm (*39*) for functional protein association networks. The nodes related to particular pathways are represented with different colors. For COX-only subset, ribonucleoproteins (red), gene expression (blue), nucleic acid binding (green) and ubiquitin binding (yellow) processes or pathways are indicated (**A**). For ND_CYTB_COX_ATP intersection, translation (red), gene expression (blue), and aerobic respiration processes or pathways are indicated (**B**).

A

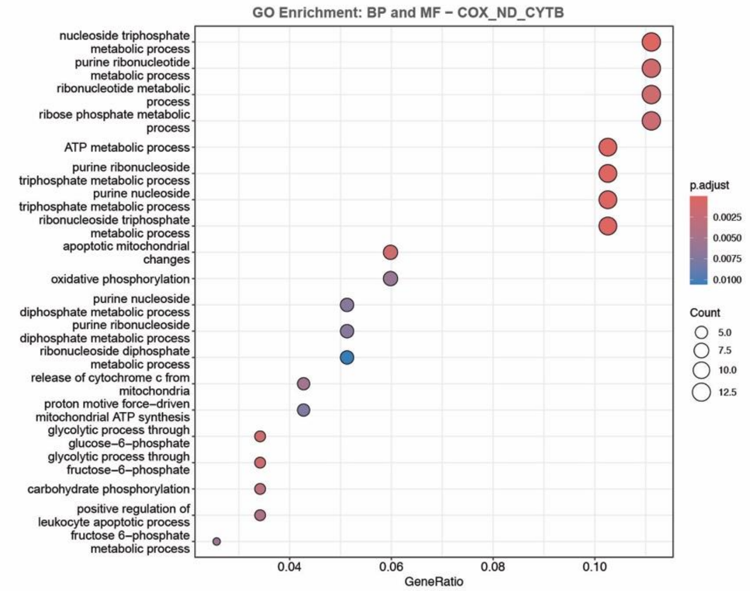
GO Enrichment: BP and MF – COX_ND_CYTB
nucleoside triphosphate metabolic process
purine ribonucleotide metabolic process
ribonucleotide metabolic process
ribose phosphate metabolic process
ATP metabolic process
purine ribonucleoside triphosphate metabolic process
purine nucleoside triphosphate metabolic process
ribonucleoside triphosphate metabolic process
apoptotic mitochondrial changes
oxidative phosphorylation
purine nucleoside diphosphate metabolic process
purine ribonucleoside diphosphate metabolic process
ribonucleoside diphosphate metabolic process
release of cytochrome c from mitochondria
proton motive force–driven mitochondrial ATP synthesis
glycolytic process through glucose–6–phosphate
glycolytic process through fructose–6–phosphate
carbohydrate phosphorylation
positive regulation of leukocyte apoptotic process
fructose 6–phosphate metabolic process
0.04
0.06
0.08
0.10
GeneRatio
p.adjust
0.0025
0.0050
0.0075
0.0100
Count
5.0
7.5
10.0
12.5

B

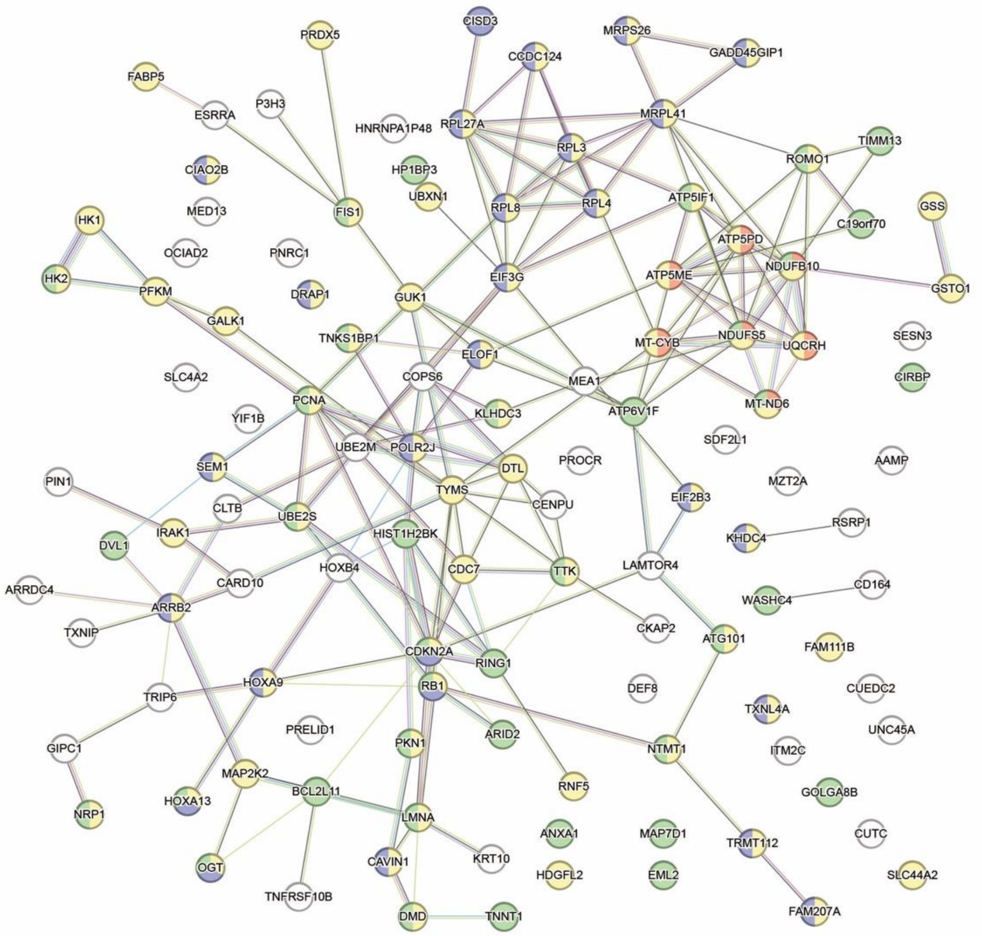
CISD3
PRDX5
MRPS26
CCDC124
GADD45GIP1
FABP5
ESRRA
P3H3
HNRNPA1P48
RPL27A
MRPL41
TIMM13
CIAO2B
HP1BP3
RPL3
ROMO1
MED13
UBXN1
FIS1
RPL8
RPL4
ATP5IF1
GSS
HK1
C19orf70
OCIAD2
PNRC1
ATP5PD
HK2
PFKM
DRAP1
GUK1
EIF3G
ATP5ME
NDUFB10
GSTO1
GALK1
TNKS1BP1
MT-CYB
NDUFS5
UQCRH
SESN3
ELOF1
SLC4A2
COPS6
MEA1
CIRBP
PCNA
KLHDC3
ATP6V1F
MT-ND6
YIF1B
UBE2M
POLR2J
SDF2L1
PIN1
SEM1
DTL
PROCR
MZT2A
AAMP
TYMS
CENPU
CLTB
EIF2B3
UBE2S
IRAK1
HIST1H2BK
KHDC4
RSRP1
DVL1
CARD10
HOXB4
CDC7
TTK
LAMTOR4
ARRDC4
ARRB2
WASHC4
CD164
TXNIP
CKAP2
ATG101
CDKN2A
FAM111B
RING1
TRIP6
HOXA9
RB1
DEF8
CUEDC2
TXNL4A
PRELID1
ARID2
UNC45A
GIPC1
PKN1
NTMT1
ITM2C
MAP2K2
RNF5
GOLGA8B
BCL2L11
HOXA13
NRP1
LMNA
ANXA1
MAP7D1
CUTC
TRMT112
KRT10
OGT
CAVIN1
HDGFL2
EML2
SLC44A2
TNFRSF10B
DMD
TNNT1
FAM207A

Fig. S5.

Characteristic response to silencing of mtDNA-encoded core component proteins complexes I, III, and IV. (**A**) Gene functional enrichment analysis by gene ontology annotation of mRNAs that significantly varied (padj<0.05) only in the exclusive intersection ND_CYTB_COX. (**B**) mRNAs forming the exclusive intersections ND_CYTB_COX subset were analyzed using the STRING algorithm for functional protein association networks. The nodes related to particular pathways are represented with different colors: OXPHOS activity (red), cellular metabolic process (yellow), gene expression (blue), and organelle organization (green).

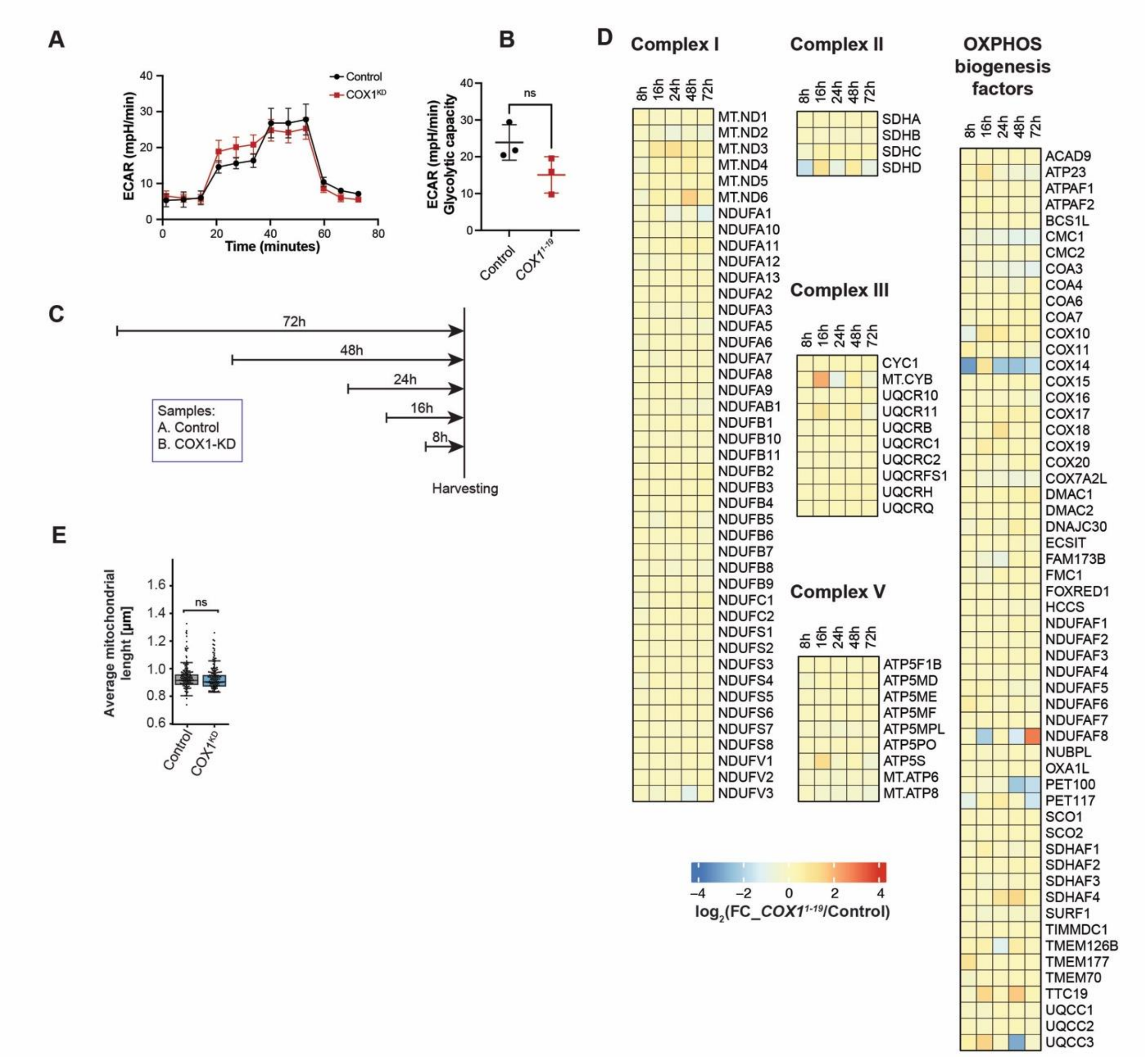

Fig. S6.

Functional mitochondrial response upon COX1 silencing. (**A**) Representative extracellular acidification rate (ECAR*)* measurements of HEK293T cells treated with pCox4-*COX1*$^{1\text{-}19}$ chimera for 72 hours. (**B**) ECAR glycolytic capacity obtained from (**A**) (ns, not significant) (n=3, mean ± SEM). (**C**) Schematic representation of the experimental design to perform COX1 silencing in HEK293T cells for different periods. Mitochondria were isolated from harvested cells and analyzed by quantitative mass spectrometry. (**D**) Heatmap of OXPHOS complexes subunits (complex I, II, III, and V) and assembly factors derived from quantitative mass spectrometric analyses (**Fig. 4N**). FC, fold change. (**E**) The mitochondrial network morphology was not affected upon COX1 silencing. After 72 hours treatment with pCox4-*COX1*$^{1\text{-}19}$ chimera, the average mitochondrial length is similar to the control (ns, not significant). Each dot represents the result of one image. p-values were calculated using unpaired t-test (mean ± SEM).

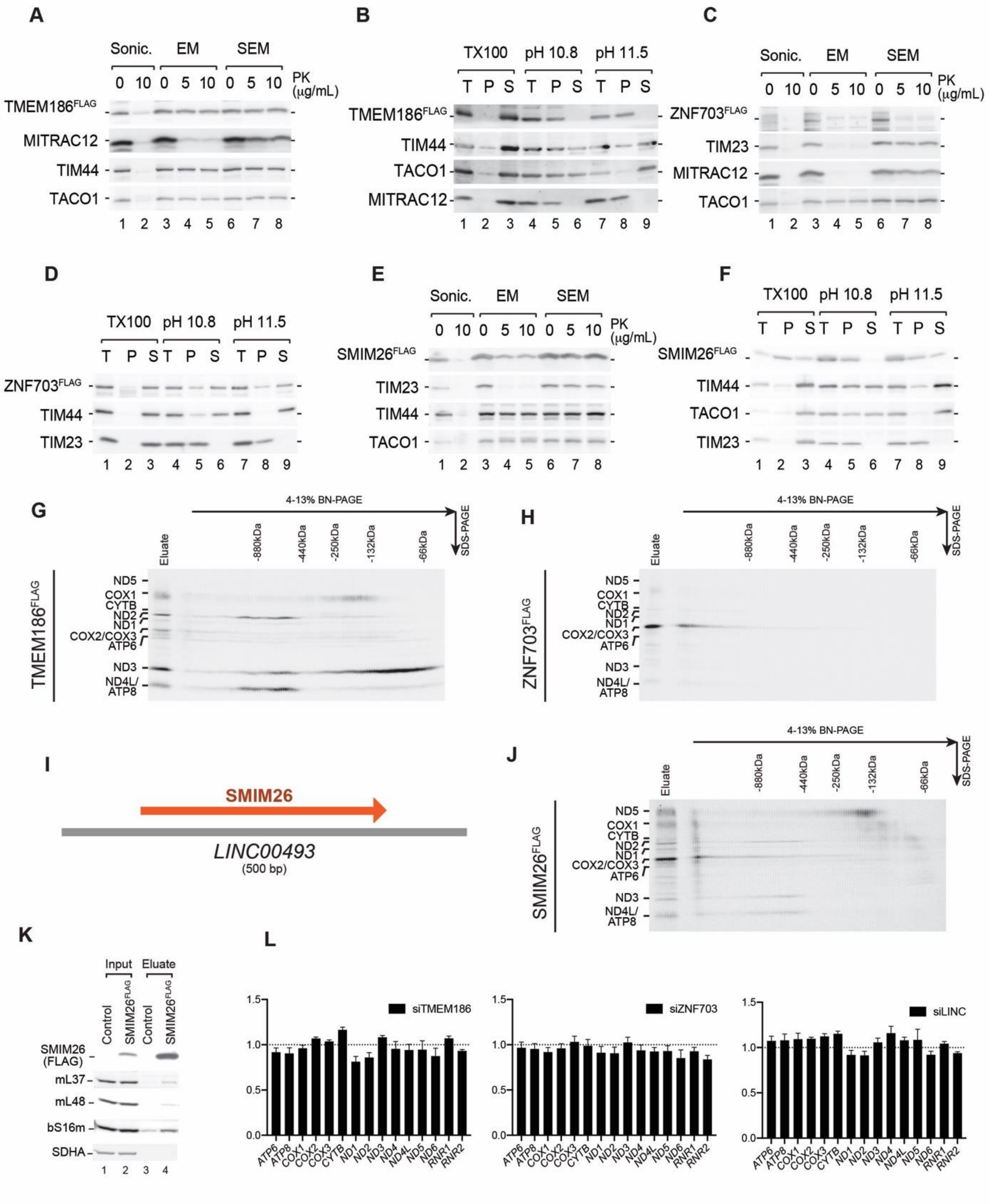
A
Sonic.
EM
SEM
PK (μg/mL)
TMEM186FLAG
MITRAC12
TIM44
TACO1
B
TX100
pH 10.8
pH 11.5
T P S
C
ZNF703FLAG
TIM23
D
E
SMIM26FLAG
F
G
4-13% BN-PAGE
SDS-PAGE
Eluate
880kDa
440kDa
250kDa
132kDa
66kDa
ND5
COX1
CYTB
ND2
ND1
COX2/COX3
ATP6
ND3
ND4L/
ATP8
H
I
SMIM26
LINC00493
(500 bp)
J
K
Input
Eluate
Control
SMIM26 (FLAG)
mL37
mL48
bS16m
SDHA
L
siTMEM186
siZNF703
siLINC
ATP6
ATP8
COX1
COX2
COX3
CYTB
ND1
ND2
ND3
ND4
ND4L
ND5
ND6
RNR1
RNR2

Fig. S7.

Characterization of identified mitochondrial biogenesis factors. (**A-F**) Proteinase K accessibility assay and organellar fractionation experiments to define the sub-mitochondrial localization of TMEM186 (A and B, respectively), ZNF703 (C and D, respectively), and SMIM26 (E and F, respectively). (**G-H**) Newly-synthesized mitochondrial proteins associated to TMEM186 (G) and ZNF703 (H). Mitochondria were purified from $TMEM186^{FLAG}$- and $ZNF703^{FLAG}$-expressing HEK293T cells, and subjected to $[^{35}S]$methionine labeling of mitochondrial translation products. Upon FLAG-immunoprecipitation, the eluates were analyzed by two-dimension (2D) BN-/SDS-PAGE and digital autoradiography. (**I**) Diagram of the long non-coding RNA LINC00493, and the open reading frame coding for SMIM26. (**J**) Newly-synthesized mitochondrial proteins associated to SMIM26 (determined as in G-H). (**K**) Mitochondria were purified from $SMIM26^{FLAG}$-expressing HEK293T cells. Upon FLAG-immunoprecipitation, the samples were analyzed by SDS-PAGE and western blotting. SMIM26 interacts with the mitochondrial ribosome. Total, 3%; eluate 100%. (**L**) RNA abundance was determined by nanoString technology in mitochondria isolated from HEK293T cells after siRNA-mediated downregulation of TMEM186, ZNF703, and *LINC00493* and compared to siNT control (dashed line)(n=4, mean ± SEM).

Table S1. Transcriptomic analyses of HEK293T cells upon treatment with peptide-morpholino chimeras for 48 hours by RNA-seq.
See also Fig. 3; fig. S3, S4, and S5

Table S2. Subsets obtained by analysis of the significantly altered transcript abundance with the UpSet intersection algorithm.
See also Fig. 3; fig. S3, S4, and S5

Table S3. Quantitative Mass Spectrometry analysis of isolated mitochondria upon ND2 and CYTB silencing in HEK293T cells for 48 hours.
See also Fig. 4

Table S4. Quantitative Mass Spectrometry analysis of isolated mitochondria upon COX1 silencing in HEK293T cells for 8, 16, 24, 48, and 72 hours.
See also Fig. 4 and fig. S6

Table S5. Quantitative Mass Spectrometry analysis of FLAG-immunoprecipitation eluates of mitochondria isolated from TMEM186^{FLAG}, ZNF703^{FLAG}, and SMIM26^{FLAG} expressing cells.
See also Fig. 5 and fig. S7

References and Notes

1. O. Rackham, A. Filipovska, Organization and expression of the mammalian mitochondrial genome. *Nat. Rev. Genet.* **23**, 606–623 (2022). doi:10.1038/s41576-022-00480-x Medline

2. B. Homberg, P. Rehling, L. D. Cruz-Zaragoza, The multifaceted mitochondrial OXA insertase. *Trends Cell Biol.* **33**, 765–772 (2023). doi:10.1016/j.tcb.2023.02.001 Medline

3. Y. Itoh, J. Andréll, A. Choi, U. Richter, P. Maiti, R. B. Best, A. Barrientos, B. J. Battersby, A. Amunts, Mechanism of membrane-tethered mitochondrial protein synthesis. *Science* **371**, 846–849 (2021). doi:10.1126/science.abe0763 Medline

4. M. Zeviani, S. Di Donato, Mitochondrial disorders. *Brain* **127**, 2153–2172 (2004). doi:10.1093/brain/awh259 Medline

5. C. B. Park, N.-G. Larsson, Mitochondrial DNA mutations in disease and aging. *J. Cell Biol.* **193**, 809–818 (2011). doi:10.1083/jcb.201010024 Medline

6. E. A. Shoubridge, A. Barrientos, Mitochondrial molecular genetics and human disease. *Hum. Mol. Genet.* **33**, R1–R2 (2024). doi:10.1093/hmg/ddae049 Medline

7. R. W. Taylor, D. M. Turnbull, Mitochondrial DNA mutations in human disease. *Nat. Rev. Genet.* **6**, 389–402 (2005). doi:10.1038/nrg1606 Medline

8. D. H. Hock, D. R. L. Robinson, D. A. Stroud, Blackout in the powerhouse: Clinical phenotypes associated with defects in the assembly of OXPHOS complexes and the mitoribosome. *Biochem. J.* **477**, 4085–4132 (2020). doi:10.1042/BCJ20190767 Medline

9. B. M. Hällberg, N.-G. Larsson, Making proteins in the powerhouse. *Cell Metab.* **20**, 226–240 (2014). doi:10.1016/j.cmet.2014.07.001 Medline

10. M. Lee, N. Matsunaga, S. Akabane, I. Yasuda, T. Ueda, N. Takeuchi-Tomita, Reconstitution of mammalian mitochondrial translation system capable of correct initiation and long polypeptide synthesis from leaderless mRNA. *Nucleic Acids Res.* **49**, 371–382 (2021). doi:10.1093/nar/gkaa1165 Medline

11. S. F. Pearce, M. Cipullo, B. Chung, I. Brierley, J. Rorbach, *Mitochondrial Gene Expression: Methods and Protocols*, M. Minczuk, J. Rorbach, Eds. (Springer, 2021), pp. 183–196.

12. C. M. Gustafsson, M. Falkenberg, N.-G. Larsson, Maintenance and Expression of Mammalian Mitochondrial DNA. *Annu. Rev. Biochem.* **85**, 133–160 (2016). doi:10.1146/annurev-biochem-060815-014402 Medline

13. P. Silva-Pinheiro, M. Minczuk, The potential of mitochondrial genome engineering. *Nat. Rev. Genet.* **23**, 199–214 (2022). doi:10.1038/s41576-021-00432-x Medline

14. R. N. Lightowlers, Mitochondrial transformation: Time for concerted action. *EMBO Rep.* **12**, 480–481 (2011). doi:10.1038/embor.2011.93 Medline

15. S.-I. Cho, K. Lim, S. Hong, J. Lee, A. Kim, C. J. Lim, S. Ryou, J. M. Lee, Y. G. Mok, E. Chung, S. Kim, S. Han, S.-M. Cho, J. Kim, E.-K. Kim, K.-H. Nam, Y. Oh, M. Choi, T. H. An, K.-J. Oh, S. Lee, H. Lee, J.-S. Kim, Engineering TALE-linked deaminases to facilitate precision adenine base editing in mitochondrial DNA. *Cell* **187**, 95–109.e26 (2024). doi:10.1016/j.cell.2023.11.035 Medline

16. B. Y. Mok, M. H. de Moraes, J. Zeng, D. E. Bosch, A. V. Kotrys, A. Raguram, F. Hsu, M. C. Radey, S. B. Peterson, V. K. Mootha, J. D. Mougous, D. R. Liu, A bacterial cytidine deaminase toxin enables CRISPR-free mitochondrial base editing. *Nature* **583**, 631–637 (2020). doi:10.1038/s41586-020-2477-4 Medline

17. B. Y. Mok, A. V. Kotrys, A. Raguram, T. P. Huang, V. K. Mootha, D. R. Liu, CRISPR-free base editors with enhanced activity and expanded targeting scope in mitochondrial and nuclear DNA. *Nat. Biotechnol.* **40**, 1378–1387 (2022). doi:10.1038/s41587-022-01256-8 Medline

18. P. Silva-Pinheiro, C. D. Mutti, L. Van Haute, C. A. Powell, P. A. Nash, K. Turner, M. Minczuk, A library of base editors for the precise ablation of all protein-coding genes in the mouse mitochondrial genome. *Nat. Biomed. Eng.* **7**, 692–703 (2023). doi:10.1038/s41551-022-00968-1 Medline

19. L. D. Cruz-Zaragoza, S. Dennerlein, A. Linden, R. Yousefi, E. Lavdovskaia, A. Aich, R. R. Falk, R. Gomkale, T. Schöndorf, M. T. Bohnsack, R. Richter-Dennerlein, H. Urlaub, P. Rehling, An *in vitro* system to silence mitochondrial gene expression. *Cell* **184**, 5824–5837.e15 (2021). doi:10.1016/j.cell.2021.09.033 Medline

20. D. Stein, E. Foster, S. B. Huang, D. Weller, J. Summerton, A specificity comparison of four antisense types: Morpholino, 2′-O-methyl RNA, DNA, and phosphorothioate DNA. *Antisense Nucleic Acid Drug Dev.* **7**, 151–157 (1997). doi:10.1089/oli.1.1997.7.151 Medline

21. T. R. Mercer, S. Neph, M. E. Dinger, J. Crawford, M. A. Smith, A.-M. J. Shearwood, E. Haugen, C. P. Bracken, O. Rackham, J. A. Stamatoyannopoulos, A. Filipovska, J. S. Mattick, The human mitochondrial transcriptome. *Cell* **146**, 645–658 (2011). doi:10.1016/j.cell.2011.06.051 Medline

22. A. Lex, N. Gehlenborg, H. Strobelt, R. Vuillemot, H. Pfister, UpSet: Visualization of Intersecting Sets. *IEEE Trans. Vis. Comput. Graph.* **20**, 1983–1992 (2014). doi:10.1109/TVCG.2014.2346248 Medline

23. M. T. Couvillion, I. C. Soto, G. Shipkovenska, L. S. Churchman, Synchronized mitochondrial and cytosolic translation programs. *Nature* **533**, 499–503 (2016). doi:10.1038/nature18015 Medline

24. I. Soto, M. Couvillion, K. G. Hansen, E. McShane, J. C. Moran, A. Barrientos, L. S. Churchman, Balanced mitochondrial and cytosolic translatomes underlie the biogenesis of human respiratory complexes. *Genome Biol.* **23**, 170 (2022). doi:10.1186/s13059-022-02732-9 Medline

25. M. Molenaars, G. E. Janssens, E. G. Williams, A. Jongejan, J. Lan, S. Rabot, F. Joly, P. D. Moerland, B. V. Schomakers, M. Lezzerini, Y. J. Liu, M. A. McCormick, B. K. Kennedy, M. van Weeghel, A. H. C. van Kampen, R. Aebersold, A. W. MacInnes, R. H. Houtkooper, A Conserved Mito-Cytosolic Translational Balance Links Two Longevity Pathways. *Cell Metab.* **31**, 549–563.e7 (2020). doi:10.1016/j.cmet.2020.01.011 Medline

26. A. Signes, E. Fernandez-Vizarra, Assembly of mammalian oxidative phosphorylation complexes I-V and supercomplexes. *Essays Biochem.* **62**, 255–270 (2018). doi:10.1042/EBC20170098 Medline

27. M. Morgenstern, C. D. Peikert, P. Lübbert, I. Suppanz, C. Klemm, O. Alka, C. Steiert, N. Naumenko, A. Schendzielorz, L. Melchionda, W. W. D. Mühlhäuser, B. Knapp, J. D. Busch, S. B. Stiller, S. Dannenmaier, C. Lindau, M. Licheva, C. Eickhorst, R. Galbusera, R. M. Zerbes, M. T. Ryan, C. Kraft, V. Kozjak-Pavlovic, F. Drepper, S. Dennerlein, S. Oeljeklaus, N. Pfanner, N. Wiedemann, B. Warscheid, Quantitative high-confidence human mitochondrial proteome and its dynamics in cellular context. *Cell Metab.* **33**, 2464–2483.e18 (2021). doi:10.1016/j.cmet.2021.11.001 Medline

28. A. Timón-Gómez, E. Nývltová, L. A. Abriata, A. J. Vila, J. Hosler, A. Barrientos, Mitochondrial cytochrome c oxidase biogenesis: Recent developments. *Semin. Cell Dev. Biol.* **76**, 163–178 (2017). doi: 10.1016/j.semcdb.2017.08.055 Medline

29. A. Suomalainen, J. Nunnari, Mitochondria at the crossroads of health and disease. *Cell* **187**, 2601–2627 (2024). doi:10.1016/j.cell.2024.04.037 Medline

30. R. Quintana-Cabrera, L. Scorrano, Determinants and outcomes of mitochondrial dynamics. *Mol. Cell* **83**, 857–876 (2023). doi:10.1016/j.molcel.2023.02.012 Medline

31. L. E. Formosa, L. Muellner-Wong, B. Reljic, A. J. Sharpe, T. D. Jackson, T. H. Beilharz, D. Stojanovski, M. Lazarou, D. A. Stroud, M. T. Ryan, Dissecting the Roles of Mitochondrial Complex I Intermediate Assembly Complex Factors in the Biogenesis of Complex I. *Cell Rep.* **31**, 107541 (2020). doi:10.1016/j.celrep.2020.107541 Medline

32. L. Sánchez-Caballero, D. M. Elurbe, F. Baertling, S. Guerrero-Castillo, M. van den Brand, J. van Strien, T. J. P. van Dam, R. Rodenburg, U. Brandt, M. A. Huynen, L. G. J. Nijtmans, TMEM70 functions in the assembly of complexes I and V. *Biochim. Biophys. Acta Bioenerg.* **1861**, 148202 (2020). doi:10.1016/j.bbabio.2020.148202 Medline

33. D. G. Holland, A. Burleigh, A. Git, M. A. Goldgraben, P. A. Perez-Mancera, S.-F. Chin, A. Hurtado, A. Bruna, H. R. Ali, W. Greenwood, M. J. Dunning, S. Samarajiwa, S. Menon, O. M. Rueda, A. G. Lynch, S. McKinney, I. O. Ellis, C. J. Eaves, J. S. Carroll, C. Curtis, S. Aparicio, C. Caldas, *ZNF703* is a common Luminal B breast cancer oncogene that differentially regulates luminal and basal progenitors in human mammary epithelium. *EMBO Mol. Med.* **3**, 167–180 (2011). doi:10.1002/emmm.201100122 Medline

34. C. Orhan, B. Bakır, N. Dalay, N. Buyru, ZNF703 is an important player in head and neck cancer. *Clin. Otolaryngol.* **44**, 1080–1086 (2019). doi:10.1111/coa.13450 Medline

35. F. Sircoulomb, N. Nicolas, A. Ferrari, P. Finetti, I. Bekhouche, E. Rousselet, A. Lonigro, J. Adélaïde, E. Baudelet, S. Esteyriès, J. Wicinski, S. Audebert, E. Charafe-Jauffret, J. Jacquemier, M. Lopez, J.-P. Borg, C. Sotiriou, C. Popovici, F. Bertucci, D. Birnbaum, M. Chaffanet, C. Ginestier, *ZNF703* gene amplification at 8p12 specifies luminal B breast cancer. *EMBO Mol. Med.* **3**, 153–166 (2011). doi:10.1002/emmm.201100121 Medline

36. D. Konina, P. Sparber, I. Viakhireva, A. Filatova, M. Skoblov, Investigation of *LINC00493/SMIM26* Gene Suggests Its Dual Functioning at mRNA and Protein Level. *Int. J. Mol. Sci.* **22**, 8477 (2021). doi:10.3390/ijms22168477 Medline

37. F. Yeasmin, N. Imamachi, T. Tanu, K. Taniue, T. Kawamura, T. Yada, N. Akimitsu, Identification and analysis of short open reading frames (sORFs) in the initially annotated noncoding RNA LINC00493 from human cells. *J. Biochem.* **169**, 421–434 (2021). doi:10.1093/jb/mvaa143 Medline

38. R. Richter-Dennerlein, S. Oeljeklaus, I. Lorenzi, C. Ronsör, B. Bareth, A. B. Schendzielorz, C. Wang, B. Warscheid, P. Rehling, S. Dennerlein, Mitochondrial Protein Synthesis Adapts to Influx of Nuclear-Encoded Protein. *Cell* **167**, 471–483.e10 (2016). doi:10.1016/j.cell.2016.09.003 Medline

39. R. C. Gentleman, V. J. Carey, D. M. Bates, B. Bolstad, M. Dettling, S. Dudoit, B. Ellis, L. Gautier, Y. Ge, J. Gentry, K. Hornik, T. Hothorn, W. Huber, S. Iacus, R. Irizarry, F. Leisch, C. Li, M. Maechler, A. J. Rossini, G. Sawitzki, C. Smith, G. Smyth, L. Tierney, J. Y. H. Yang, J. Zhang, Bioconductor: Open software development for computational biology and bioinformatics. *Genome Biol.* **5**, R80 (2004). doi:10.1186/gb-2004-5-10-r80 Medline

40. W. Huber, V. J. Carey, R. Gentleman, S. Anders, M. Carlson, B. S. Carvalho, H. C. Bravo, S. Davis, L. Gatto, T. Girke, R. Gottardo, F. Hahne, K. D. Hansen, R. A. Irizarry, M. Lawrence, M. I. Love, J. MacDonald, V. Obenchain, A. K. Oleś, H. Pagès, A. Reyes, P. Shannon, G. K. Smyth, D. Tenenbaum, L. Waldron, M. Morgan, Orchestrating high-throughput genomic analysis with Bioconductor. *Nat. Methods* **12**, 115–121 (2015). doi:10.1038/nmeth.3252 Medline

41. M. I. Love, W. Huber, S. Anders, Moderated estimation of fold change and dispersion for RNA-seq data with DESeq2. *Genome Biol.* **15**, 550 (2014). doi:10.1186/s13059-014-0550-8 Medline

42. M. E. Ritchie, B. Phipson, D. Wu, Y. Hu, C. W. Law, W. Shi, G. K. Smyth, limma powers differential expression analyses for RNA-sequencing and microarray studies. *Nucleic Acids Res.* **43**, e47–e47 (2015). doi:10.1093/nar/gkv007 Medline

43. G. Yu, L.-G. Wang, Y. Han, Q.-Y. He, clusterProfiler: An R package for comparing biological themes among gene clusters. *OMICS* **16**, 284–287 (2012). doi:10.1089/omi.2011.0118 Medline

44. J. R. Conway, A. Lex, N. Gehlenborg, UpSetR: An R package for the visualization of intersecting sets and their properties. *Bioinformatics* **33**, 2938–2940 (2017). doi:10.1093/bioinformatics/btx364 Medline

45. Y. Perez-Riverol, C. Bandla, D. J. Kundu, S. Kamatchinathan, J. Bai, S. Hewapathirana, N. S. John, A. Prakash, M. Walzer, S. Wang, J. A. Vizcaíno, The PRIDE database at 20 years: 2025 update. *Nucleic Acids Res.* **53**, D543–D553 (2025). doi:10.1093/nar/gkae1011 Medline

46. J. Bender, aretaon/silencing-mito-genes-analysis: Initial release for paper publication, Version v1.0.0, Zenodo (2025); https://doi.org/10.5281/zenodo.15241939

47. A. Zheenbekova, M. Lidschreiber, cramerlab/Silencing-mitochondrial-gene-expression-in-living-cells: v1, Version v1, Zenodo (2025); https://doi.org/10.5281/zenodo.15260712

48. R. Gomkale, L. D. Cruz-Zaragoza, I. Suppanz, B. Guiard, J. Montoya, S. Callegari, D. Pacheu-Grau, B. Warscheid, P. Rehling, Defining the Substrate Spectrum of the TIM22 Complex Identifies Pyruvate Carrier Subunits as Unconventional Cargos. *Curr. Biol.* **30**, 1119–1127.e5 (2020). doi:10.1016/j.cub.2020.01.024 Medline

49. C. D. Peikert, J. Mani, M. Morgenstern, S. Käser, B. Knapp, C. Wenger, A. Harsman, S. Oeljeklaus, A. Schneider, B. Warscheid, Charting organellar importomes by quantitative mass spectrometry. *Nat. Commun.* **13**, 15272 Medline (2017). doi:0.1038/ncomms15272

50. S. Tyanova, T. Temu, P. Sinitcyn, A. Carlson, M. Y. Hein, T. Geiger, M. Mann, J. Cox, The Perseus computational platform for comprehensive analysis of (prote)omics data. *Nat. Methods* **13**, 731–740 (2016). doi:10.1038/nmeth.3901 Medline

51. V. Todorov, M. Templ, P. Filzmoser, Detection of multivariate outliers in business survey data with incomplete information. *Adv. Data Anal. Classif.* **5**, 37–56 (2011). doi:10.1007/s11634-010-0075-2

52. F. Del Carratore, A. Jankevics, R. Eisinga, T. Heskes, F. Hong, R. Breitling, RankProd 2.0: A refactored bioconductor package for detecting differentially expressed features in molecular profiling datasets. *Bioinformatics* **33**, 2774–2775 (2017). doi:10.1093/bioinformatics/btx292 Medline

53. W. Huber, A. von Heydebreck, H. Sültmann, A. Poustka, M. Vingron, Variance stabilization applied to microarray data calibration and to the quantification of differential expression. *Bioinformatics* **18**, S96–S104 (2002). doi:10.1093/bioinformatics/18.suppl_1.S96 Medline

54. E. Höck, T.-O. Buchholz, A. Brachmann, F. Jug, A. Freytag, L. Karlinsky, T. Michaeli, K. Nishino, Eds., (Springer, 2023), pp. 503–518.

55. M. Weigert *et al.*, Star-convex Polyhedra for 3D Object Detection and Segmentation in Microscopy. 2020 IEEE Winter Conference on Applications of Computer Vision (WACV). 3655–3662 (2020).

56. S. van der Walt, J. L. Schönberger, J. Nunez-Iglesias, F. Boulogne, J. D. Warner, N. Yager, E. Gouillart, T. Yu, scikit-image contributors, scikit-image: Image processing in Python. *PeerJ* **2**, e453 (2014). doi:10.7717/peerj.453 Medline
